# Coengineering Vapor-Deposited Polycationic Microenvironments and Immobilization Strategy to Enhance β‑Galactosidase Kinetics at Acidic pH

**DOI:** 10.1021/acsomega.6c02352

**Published:** 2026-08-17

**Authors:** Felicia Fianu, Junxing Chen, Babak Faraji-Gougerdchi, Huida Duan, Haibo Huang, Rong Yang, Wei Sun, Yifan Cheng

**Affiliations:** † Virginia Polytechnic Institute and State University, Department of Food Science and Technology, Blacksburg, Virginia 24061, United States; ‡ Virginia Polytechnic Institute and State University, Department of Biochemistry, Blacksburg, Virginia 24061, United States; § 5922 Cornell University, Smith School of Chemical and Biomolecular Engineering, Ithaca, New York 14853, United States

## Abstract

The industrial application of enzymes, such as β-galactosidase (LacZ) for processing acid whey, is often constrained by their poor activity at acidic pH. This work addresses this limitation by covalently immobilizing LacZ on copolymer thin films synthesized by initiated chemical vapor deposition (iCVD) containing 2-(dimethylamino)­ethyl methacrylate (DMAEMA), which becomes positively charged at acidic pH. Chemically analogous self-assembled monolayer (SAM) supports served as a solution-synthesized benchmark. We systematically investigated immobilization strategies—random immobilization (RI) vs directed immobilization (DI, via SpyCatcher/SpyTag)—and support properties (polycationic content, cross-linking degree, support thickness, and synthesis route: iCVD vs SAM), quantifying their effects on LacZ activity and kinetic parameters. Generally, our results indicate that LacZ immobilized via DI outperformed RI across support properties under wide pHs (4–8), mainly by enhancing the LacZ’s intrinsic turnover number (*k*_cat_) rather than by reducing Michaelis constant (*K*_m_). For both DI and RI, cationic moieties in the supports were crucial for preserving LacZ activity under stronger acid stress (pH 4); moreover, under DI, *k*_cat_ scaled with DMAEMA molar content, consistent with a proposed electrostatic shielding mechanism that may reduce local proton exposure that would otherwise depress *k*_cat_. Additionally, holding polycationic content constant (25 mol %), the iCVD films (∼200 nm) exhibited an approximately 8-fold higher normalized initial rate relative to SAM films at pH 4, but this enhancement was detectable only for DI, not RI. The optimal formulation depended on the performance metric, with distinct formulations maximizing *k*_cat_ and catalytic efficiency (*k*_cat_/*K*_m_). These findings provide mechanistic insights into how immobilization strategy and the local microenvironment jointly affect LacZ kinetics, demonstrating that coengineering both may achieve superior enzyme performance under challenging reaction conditions.

## Graphical abstract

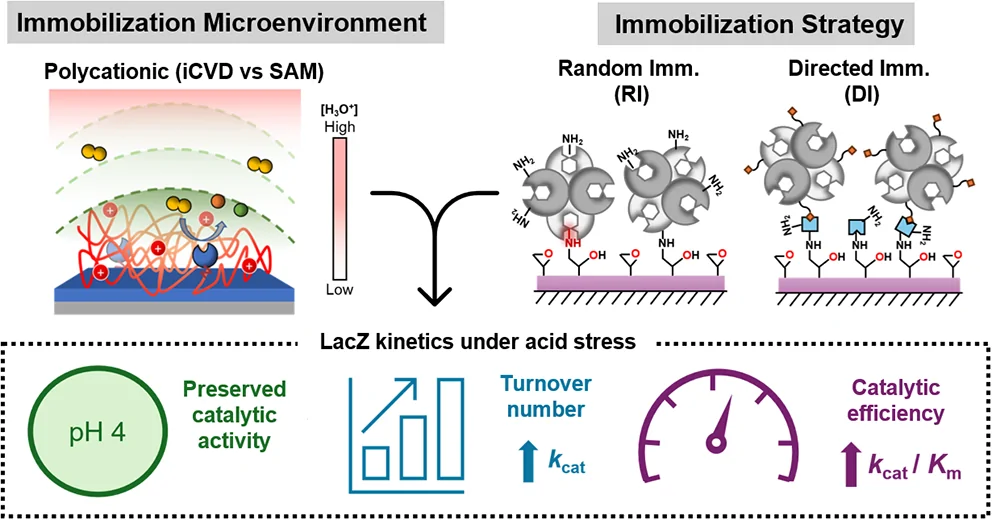

## 1 Introduction

Enzyme immobilization has emerged as a pivotal strategy for enhancing enzyme performance in various industrial and biotechnological processes.^1,2^ By immobilizing enzymes onto solid supports, significant advantages can be realized, including reusability of enzymes, improved operational stability under harsh conditions (e.g., extreme temperatures and acidic or alkaline pH), simplified separation and purification of reaction products, and reduced process costs.^2–6^ Despite these notable advantages, enzyme immobilization can also negatively impact enzyme activity due to conformational strain and restricted substrate accessibility caused by unfavorable enzyme–support interactions.^3,7–10^ Designing and engineering immobilization supports that preserve enzyme activity remain a long-standing challenge.^4,8^

Surface engineering techniques like initiated chemical vapor deposition (iCVD) and self-assembled monolayer (SAM) offer precise control over polymer surface chemistry and structure, enabling uniform support coatings with tailored chemical functionalities. This precise control is particularly beneficial in immobilization scenarios requiring uniform enzyme distribution, controlled surface chemistry, and consistent microenvironmental conditions across the enzyme–support interface.^11–13^ However, iCVD and SAM differ in their synthesis routes: SAM is a typical solution-based approach for preparing monolayer coatings with well-defined terminal functional groups, suitable for enzyme immobilization with highly specific functional groups.^14–18^ Nonetheless, its application may be limited by solvent–substrate incompatibilities and formation of certain defects such as multilayers and/or aggregates.^19^ In contrast, iCVD is an all-dry, solvent-free, vapor-phase polymerization method that operates at near-ambient substrate temperatures (typically 20–40 °C) and yields highly conformal polymer coatings with well-controlled thickness (from <5 nm to 15 μm).^11,12,18,20,21^ Accordingly, iCVD enables direct deposition onto solvent- or heat-sensitive substrates, including a broad range of biomaterials (e.g., hydrogel films, textiles, and implantable device components).^22^ A direct comparison between iCVD- and SAM-based immobilization supports, particularly their effects on enzyme kinetics across common processing pHs, can yield mechanistic insight into enzyme–support interactions and inform synthesis-route selection for various applications.

In addition to the properties of immobilization supports, enzyme performance is strongly dependent on the immobilization strategy (e.g., adsorption, covalent attachment, entrapment/encapsulation, or carrier-free cross-linking).^23^ This is because these choices govern enzyme orientation/multipoint attachment, local microenvironment, and mass-transfer limitations that collectively determine enzymatic kinetic parameters and operational stability.^23^ In this work, we focus on covalent attachment because robust covalent linkages minimize enzyme leaching or detachment during repeated operations, improving the sustainability of enzymatic catalysis. Random immobilization (RI), such as conjugation of protein nucleophiles (typically amines) to the epoxide rings on the support, is straightforward but can yield heterogeneous enzyme orientations that obstruct the active site or perturb enzyme conformation.^24–27^ In contrast, directed (or site-specific) immobilization (DI) methods, such as via SpyCatcher/SpyTag, enable single-site conjugation via a spontaneous covalent isopeptide bond (Lys-Asp), yielding more uniform orientation and reducing unfavorable enzyme–support interactions that can impose conformational strain and/or obstruct the substrate access to the active site.^28–30^

Despite extensive studies, the interplay between immobilization microenvironment and immobilization strategy on catalytic behavior remains poorly understood, particularly under industrially relevant, suboptimal pH conditions.^5,31–34^ Thus, this study aims to systematically evaluate the combined effects of support synthesis techniques (iCVD and SAM), polymer chemistry (especially inclusion of polycationic moieties and cross-linkers, which will enhance electrostatic stabilization and structural rigidity, respectively), and immobilization strategies (RI vs DI) on the catalytic performance of β-galactosidase (LacZ), a well-studied model system, especially on the key kinetic parameters: Michaelis constant (*K*_m_), turnover number (*k*_cat_), and catalytic efficiency (*k*_cat_/*K*_m_). LacZ is an enzyme that catalyzes the hydrolysis of lactose into glucose and galactose and is most active under neutral to slightly basic conditions. Depending on its microbial source, its optimal pH ranges from about 4 to 8 (with many mesophilic forms operating best near pH 6.5–7.5), and its optimal temperature typically lies between 35 and 70 °C.^35–37^ By exposing LacZ to various degrees of acid stress (pH 4–8), including strong acid stress relevant to acid whey processing (pH ∼4), we aim not only to elucidate potential interplay between immobilization microenvironment and immobilization strategy across pH but also to assess whether coengineering both can further improve performance for enzyme-driven industrial applications.

## 2 Materials and Methods

### 2.1 Nomenclature

iCVD monomers: GMA = glycidyl methacrylate, DMAEMA = 2-(dimethylamino)­ethyl methacrylate, and EGDMA = ethylene glycol dimethacrylate. SAM monomers: GPTMS = 3-glycidyloxypropyl trimethoxysilane, DMAPTMS = 3-(dimethylamino)­propyl trimethoxysilane.

We adopted the following polymer naming convention for this study: We use “G” for the epoxide monomers (GMA of iCVD and GPTMS of SAM), “D” for the tertiary amine monomers (DMAEMA of iCVD and DMAPTMS of SAM), and “E” for the cross-linker EGDMA of iCVD. At the beginning of a name, lowercase “p” signifies iCVD-synthesized polymer thin films, whereas uppercase “S” signifies SAM monolayers.

### 2.2 Chemical Reagents

All precursors, including iCVD monomers (GMA, EGDMA, DMAEMA) and iCVD initiator (TBPO), were purchased from Sigma-Aldrich Inc. (St. Louis, MO, USA) and SAMs (GPTMS, DMAPTMS) from Fisher Scientific Company LLC (3970 Johns Creek Ct Ste 500 Suwanee, Georgia 30024–1297, USA) and used without further purification. Specifically, the purities of the iCVD monomers are GMA (97.0%, hydroquinone monomethyl ether ∼0.01% as stabilizer), EGDMA (98%, 90–110 ppm monomethyl ether hydroquinone as antipolymerization agent), DMAEMA (98%, 700–1000 ppm monomethyl ether hydroquinone as inhibitor); iCVD initiator TBPO (98%), SAMs monomers, GPTMS (98%, TCI America), and DMAPTMS (96%, TCI America). Phosphate-buffered saline (PBS) (10×) pH 7.4 was purchased from Fisher Scientific and diluted to 1× for use. The protein quantification assay kits, including the Pierce bicinchoninic acid (BCA) assay kit (assay range: 20–2000 μg/mL) and the Bradford assay kit (assay range: 1–1500 μg/mL), were purchased from Thermo Fisher Scientific (Waltham, MA, USA). 2-Nitrophenyl β-d-galactopyranoside (≥98% enzymatic, ONPG) and 2-Nitrophenol (98%, ONP) were purchased from Sigma-Aldrich Inc. (St. Louis, MO, USA).

### 2.3 Fabrication of Immobilization Support Using iCVD

iCVD-based supports comprised the following monomers with varying compositions: GMA (which bears an epoxide for covalent conjugation with enzyme/protein), DMAEMA (which bears tertiary amines for local pH regulation), and EGDMA (which serves as a cross-linker to stabilize the immobilization support). Tert-butyl peroxide served as the initiator, consistent with typical iCVD polymerization processes.^20,38^ The precursors for the immobilization support with specific surface chemistries were vaporized and introduced into the iCVD chamber at a total flow rate ranging from 3 to 5 sccm (1 sccm = 1 standard cm^3^/min = 1.67 × 10^–8^ m^3^/s). The flow rate of TBPO, the stage temperature, and the total process pressure were maintained at 0.6 sccm, 35 °C, and 0.6 Torr (1 Torr = 133.33 Pa), respectively, for all depositions. The gas flow rates, *P*_m_/*P*_sat_, and the resulted deposition rate for iCVD are summarized in Tables S1 and S2, respectively. The polymerization reaction was initiated by resistively heating a filament array to a temperature of 220 °C to form primary free radicals from TBPO. Polystyrene 96-well plates (Corning 96 Well Eia/Ria Assay Microp) were used as substrates for LacZ immobilization and enzyme kinetic experiments. The 96-well plates were placed on a custom-made aluminum plate, which fits well with the bottom of the 96-well plates, to facilitate heat transfer to the reactor cooling stage during the coating process. During each deposition, a silicon substrate placed atop the 96-well plate was used for *in situ* film thickness monitoring via an interferometer equipped with a He–Ne laser (350–700 nm, Thorlabs Inc., Newton, NJ, USA). For all depositions, we target a coating thickness of 50 nm on the Si atop the 96-well plates with a local temperature of ∼65 °C (pressure saturation ratio *P*_m_/*P*_sat_∼0.01). The temperature measured at the bottom of the well was ∼50 °C (*P*_m_/*P*_sat_∼0.03). Based on a quadratic relationship between the deposition rate and the monomer gas when *P*_m_/*P*_sat_ is small (<0.04 for acrylates),^39^ this temperature corresponds to an estimated film thickness of approximately 200 nm on the bottom surface of each well.

### 2.4 Fabrication of Self-Assembled Monolayers (SAMs)

SAM precursors, GPTMS and DMAPTMS, were selected to match the critical functional groups, GMA and DMAEMA, in the iCVD supports. To facilitate the grafting of SAMs onto 96-well plates and silicon wafers, both substrates were pretreated with oxygen plasma (3 min, 45 W, 500–900 mTorr; Harrick Plasma Inc., NY, USA) before exposure to SAM precursors. This step introduced hydroxyl functional groups onto the microplate surface, enabling covalent −O–Si–O– bonding of SAMs with hydroxyl groups via silanization reactions. GPTMS and DMAPTMS working solutions (1%v/v in ethanol) were prepared separately by stirring at 650 rpm at 20 °C and atmospheric pressure for 1 h. The working solutions were combined to yield a GPTMS-DMAPTMS mixture containing 75 mol % GPTMS and 25 mol % DMAPTMS, matching the composition of pGD(25). Plasma-treated substrates (96-well plate and silicon wafer) were coated by immersion in SAM precursor solutions for 18 h at 20 °C. The SAMs-functionalized substates were then rinsed with ethanol, distilled water, and dried, as described previously.^40^

### 2.5 Characterizations of Immobilization Supports

All characterizations were done immediately after synthesis or stored under vacuum until measurement to minimize exposure to moisture and oxygen. The thickness of the as-deposited iCVD films was measured *ex situ* on the witness silicon wafer placed atop each 96-well plate using a J.A. Woollam α-SE Spectroscopic Ellipsometer (Lincoln, NE, USA) at incident angles of 65°, 70°, and 75°. The film thickness was fitted using the Cauchy–Urbach model. Means of thickness values from three spots on each sample were reported.

Fourier-transform infrared (FTIR, Nicolet iS50 Model; Thermo Scientific, Austin, TX, USA) was used to confirm the presence and preservation of characteristic functional groups in the iCVD-synthesized polymer films. A mercury-cadmium-telluride (MCT) detector cooled with liquid nitrogen was used over 600–4000 cm^–1^ (resolution: 4 cm^–1^, averaged over 128 scans). Compositions of the iCVD copolymer were assessed in transmission mode. For characterizing SAMs and identification of epoxide ring-opening signatures, attenuated total reflectance (ATR)-FTIR spectra were acquired on the same instrument equipped with a grazing angle ATR accessory (VariGATR, GAT–V-N18; Harrick Scientific Products, NY) at a fixed incident angle of 60°. Baseline correction was applied by subtracting a background spectrum obtained from an uncoated Si wafer substrate. Each FTIR spectrum was normalized using the absorbance intensity of the highest peak within that spectrum.

X-ray photoelectron spectroscopy (XPS; PHI Quantera SXM) was conducted with a monochromatized Al K_α_ (1486.7 eV) X-ray source with a highly focused beam. High-resolution XPS spectra of C 1s and O 1s core electrons were obtained using a pass energy of 26 eV and a step size of 0.05 eV. Prior to analysis, samples were stored under vacuum overnight.

Surface topography was characterized by using atomic force microscopy (Jupiter XR, Oxford Instruments) operating in tapping mode. Images were collected over 5 μm × 5 μm areas at 0.5 Hz using a PPP-NCSTR cantilever (*k* ≈ 29 N m^–1^; NANOSENSORS, Switzerland) mounted in a gas-phase holder under ambient conditions. Prior to imaging, the cantilever’s spring constant and resonance frequency (∼180 kHz) were calibrated in air via the instrument’s thermal “BlueDrive” routine. A scan speed of 6.26 μm s^–1^ was used, and each scan was composed of 512 × 512 pixels. RMS roughness was calculated from the height maps using Gwyddion (version 2.63).

### 2.6 Bioengineering of Enzymes

LacZ-WT, LacZ-SpyTag (LacZ-ST), and SpyCatcher (SC) were prepared as previously reported.^41^ Briefly, the LacZ gene (*Escherichia coli* β-galactosidase; UniProt ID: P00722) was cloned into the pBAD vector to generate pBAD-LacZ-WT, while LacZ-ST was generated by fusing the SpyTag sequence with the GS linker to the N-terminus of LacZ. The SC sequence (GenBank: AFD50637.1) was codon-optimized, synthesized, and cloned into the pBAD vector to generate pBAD-SC. All constructs contained a 6 × His tag, and primer sequences are provided in Table S4. For protein expression, pBAD-LacZ-WT, pBAD-LacZ-SpyTag, and pBAD-SC were separately transformed into chemically competent *E. coli* DH10B cells and selected on LB-Amp100 agar plates. Single colonies were expanded in LB-Amp100 medium and induced at OD600 = 0.4–0.6 with 0.2% (w/v) l-arabinose. LacZ-WT and LacZ-ST were expressed at 37 °C for 24 h, whereas SC was expressed at 18 °C for 24 h. Cells were harvested, lysed by sonication, and clarified lysates were purified using Ni-NTA affinity chromatography. Purified proteins were buffer-exchanged into storage buffer, flash-frozen, and stored at −80 °C until use.

### 2.7 Protein/Enzyme Immobilization

For RI, 200 μL of LacZ solution (0.06 mg/mL, or 1.0 × 10^–4^ mM on a LacZ tetramer, LacZ_4_, basis) was dispensed into various 96-well plates with predetermined supports. The plate was incubated statically at 37 °C for 24 h to facilitate the covalent immobilization of LacZ-WT onto the coated surface. In contrast, DI entails three steps: (i) random immobilization of SC, (ii) blocking by glycine, and (iii) immobilization of LacZ-ST via SC/ST binding. The role of SC in DI was to control enzyme orientation/conjugation sites during immobilization. First, 200 μL of SC solution (0.05 mg/mL, or 2.8 × 10^–4^ mM) was randomly immobilized under the same conditions as LacZ-WT. Then, the samples underwent blocking with 1.0 M glycine to neutralize unreacted epoxide on the immobilization.^42^ Finally, the LacZ-ST (0.06 mg/mL, or 1.0 × 10^–4^ mM of LacZ_4_) was bonded to the surface-bound SC by shaking incubation at 30 rpm, 25 °C for 2 h.^30^ Following the incubation periods, any residual enzyme solution in the wells was removed, and the wells were rinsed twice with 1× PBS to eliminate any unbound/loosely adsorbed LacZ. Immobilized LacZ was used immediately for activity and protein quantification.

The expressed free protein/enzyme’s concentration was measured using Bradford assay (linear range: 1–1500 μg/mL) by following the manufacturer’s procedures. The quantification of the immobilized LacZ was performed using the BCA assay (20–2000 μg/mL) following the manufacturer’s standard operating procedures. Briefly, a 25 μL aliquot of 0.1 M phosphate buffer (pH 7) was added to the wells with immobilized enzymes (LacZ-WT (RI); SC and LacZ-ST (DI)) on a 96-well plate, followed by adding 200 μL of the BCA working reagent and mixing thoroughly on a plate shaker for 30 s. This was followed by incubation at 37 °C for 30 min and measuring absorbance at 562 nm using a microplate reader (Synergy H1, Biotek). For DI, BCA assays were performed on separate samples to determine stepwise yield: SC was first immobilized and quantified, followed by immobilization and quantification of LacZ-ST. The immobilization yield (IY) of proteins was determined using the formula

1 $$\mathrm{IY}=100\times C_{f}/C_{i}\%$$

where *C*_i_ denotes LacZ concentration in solution before immobilization, and *C*_f_ denotes the equivalent solution concentration of the immobilized LacZ measured by using the Bradford or BCA assay, respectively.

### 2.8 Determination of Activity and Kinetic Parameters

The activities of the LacZ in solution (free LacZ) and immobilized LacZ were assayed at 37 °C using ONPG as substrate. The ONPG assay was adjusted to specific pH values using 0.1 M lactate buffers (pH 4, pH 5) and 0.1 M phosphate buffers (pH 6, pH 7, pH 8). Immobilized LacZ on a polymer-coated 96-well plate for each activity test was preheated to the experimental temperature of 37 °C before the addition of substrate solutions. Five different ONPG concentrations (0, 0.66, 1.66, 3.32, 16.6 mM) in each of the above pH buffers were added to the free and immobilized LacZ for the conversion of ONPG to *o*-nitrophenol (ONP). Absorbance at 420 nm was recorded using a microplate reader (Synergy H1) for 2 min at pH 6–8 and for 5 min at pH 4, 5 to compensate for the lower activity at acidic pH. Additionally, standard curves were made using commercially obtained ONP (0–4 mM) at pH 4–8 to confirm that it can be reliably detected at 420 nm under these pHs despite differences in ONP ionization as a function of pH (Figure S7).

Normalized initial rate (*v*_0_^*^), defined as the initial rate of ONP generation normalized by the LacZ monomer concentration in the system, was used to compare the activity of free and immobilized LacZ across different samples. The LacZ activity and *v*_0_^*^ are used interchangeably in this work. ONP concentrations were calculated from 420 nm absorbance using the pH 7 calibration curve without correction for the pH-dependent ionization of ONP. Accordingly, comparisons among samples assayed at the same pH remain valid, whereas direct quantitative comparisons across pH conditions should be avoided. A nonlinear regression using the Michaelis–Menten model was conducted in RStudio to derive kinetic parameters such as Michaelis–Menten constant (*K*_m_), turnover number (*k*_cat_), and catalytic efficiency (*k*_cat_/*K*_m_) of LacZ.

### 2.9 Statistical Analysis

All experiments were performed at least in triplicate (*N* ≥ 3). Differences among treatments were evaluated by one-way ANOVA followed by Tukey’s HSD post hoc test (α = 0.05), with significant differences denoted using a compact letter display. Data were visualized in OriginLab, and statistical analyses were performed in RStudio.

## 3 Results and Discussion

### 3.1 Characterization of Immobilization Supports

We first confirmed the chemical structures and molar compositions of the iCVD-synthesized thin films and the corresponding SAMs using FTIR spectroscopy (Figure 1). The iCVD samples include homopolymers, pG, pE, and pD, and copolymers, pGE(25), pGD(25), pGD(65), and pGDE; SAM samples include homopolymers, SD and SG, and an SAM copolymer, SGD(25). FTIR spectra confirmed the presence of characteristic functional groups responsible for enzyme immobilization and pH buffering (Figure 1). The epoxide peak at 910 cm^–1^ (ring deformation of GMA or GPTMS) and tertiary amine C–H stretch at 2770 cm^–1^ (DMAEMA or DMAPTMS) were retained in all relevant copolymers.^43^ The C–N stretch (1020 cm^–1^,^44^ denoted by the red asterisk in Figure 1b) and the N–CH_3_ deformation vibration (968 cm^–1^,^45^ denoted by the red arrow in Figure 1b) might also serve as diagnostic bands and were visible for pD; however, due to its medium-to-low intensity and overlaps with other vibrations in the fingerprint region, the C–H stretch band (2770 cm^–1^) was preferred for semiquantitative “D” content estimation in this work. The strong peak at 1730 cm^–1^ is attributed to the carbonyl (C=O) stretch from methacrylate groups, which were present in all iCVD polymers but absent in SAM coatings, as expected. A weak peak in the 1660–1640 cm^–1^ range postpolymerization, marked by the black asterisk with dashed line in Figure 1b, was observed only in iCVD polymers containing a cross-linker “E” (see pE, pGE), which can be attributed to the residual C=C stretching of pendent methacrylate vinyl bonds,^46^ suggesting EGDMA were not fully cross-linked; aside from the ones with “E,” the C=C was almost absent in iCVD polymers, indicating successful polymerization. Unlike pE and pGE, the pGDE spectrum lacked an observable residual C=C and instead exhibited a distinct absorption centered at ∼1600 cm^–1^ (denoted by the black arrow in Figure 1b). This region is diagnostic for the asymmetric stretch of carboxylate ions^47^ (typically 1550–1650 cm^–1^), likely arising from partial hydrolysis of EGDMA ester bonds and stabilized by proton transfer to DMAEMA tertiary amines to form internal ion pairs (COO^–^···H–N^+^(CH_3_)_3_).

**Figure 1:**
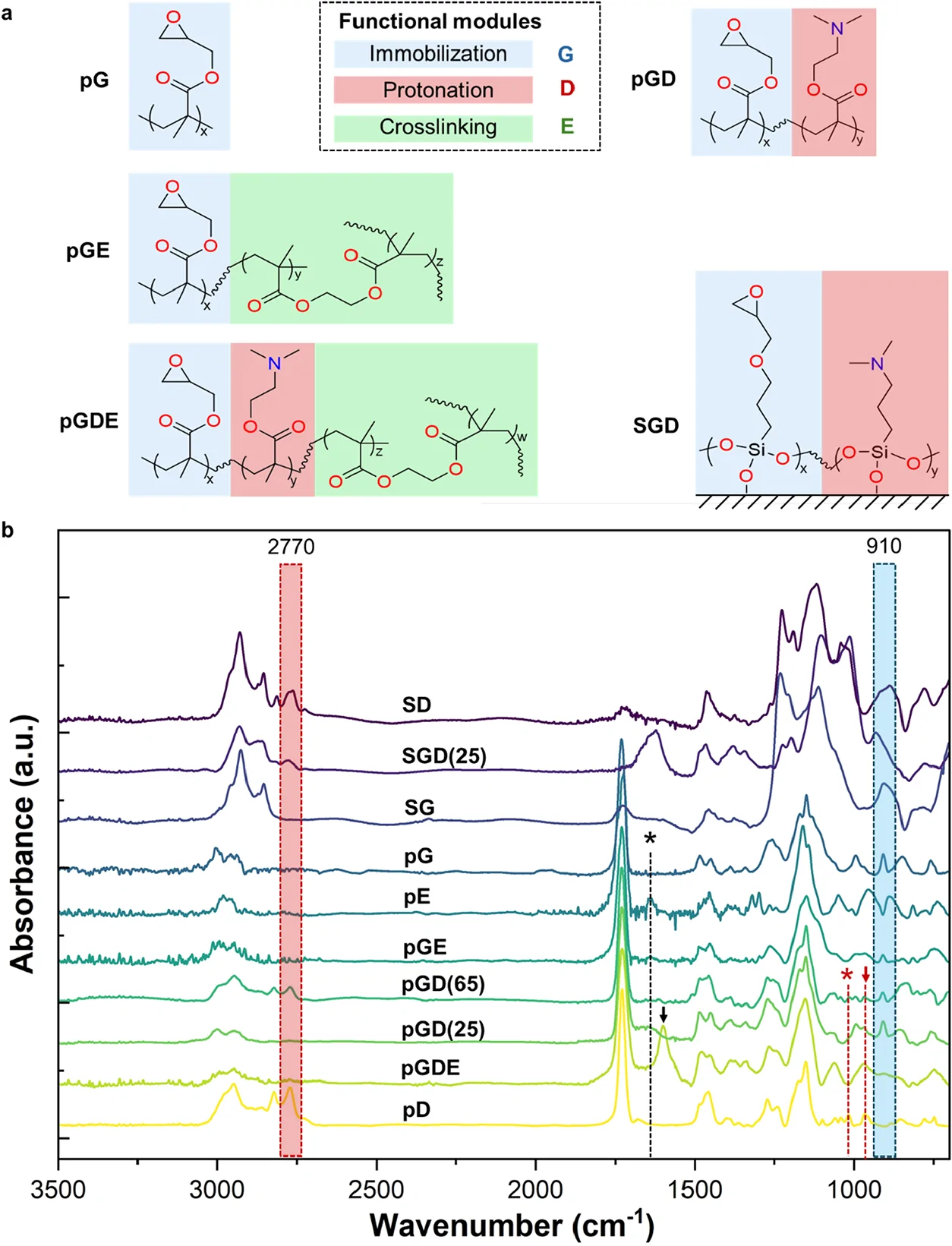
FTIR characterization of iCVD-synthesized thin films and solution-deposited SAMs. (a) Structures and functional modules of various iCVD and SAMs supports used for enzyme immobilization. (b) FTIR characterization of these polymer supports and confirmation of the critical functional groups, epoxide (blue box) and tertiary amine (red box). Numbers in parentheses represent mol % of D in the copolymer samples.

Relative intensities of the epoxide and tertiary amine peaks reflected the targeted feed ratios of monomers (Tables S1–S3). For example, pGD(65) exhibited stronger tertiary amine absorption and weaker epoxide intensity than pGD(25), consistent with increased DMAEMA incorporation. Epoxide contents (G) in pGD(25), pGD(65), and pGDE were 76, 35, and 49 mol %, respectively.

Water contact angle measurements on iCVD-synthesized samples further confirmed the tunable surface chemical feature. pD was strongly hydrophilic, and the film visibly swelled upon contact with the water droplet and resulted in low apparent water contact angles (32.8° ± 1.8°, mean ± SD), consistent with the high polarity and water solubility of pD in the absence of cross-linking. pG was more hydrophobic (70.9 ± 0.6°). Unexpectedly, after copolymerizing, pGD(25) turned out to be more hydrophobic (96.2 ± 6.6°) than either constituent. The higher apparent hydrophobicity of pGD(25) relative to pG suggests that DMAEMA-derived polarity is not fully accessible at the interface under the measurement conditions, possibly due to cross-linking between G (epoxide) and D (tertiary amine or quaternary ammonium), restricting chain mobility and burying the polar groups. In fact, increasing D from 25 to 65% appeared to reduce the water contact angle from 96.2° to 81.2°. The other polymer compositions, pE, pGE, and pGDE, displayed intermediate hydrophobicity, with contact angles between 70° and 85°, showing no significant differences (*p* > 0.05) (Figure S1a).

Nanoindentation showed that the iCVD coatings had elastic moduli of ∼2–19 GPa (Figure S1b), consistent with iCVD polymer thin films under ambient dry conditions.^48,49^ The film thicknesses and corresponding indentation depths are summarized in Table S11. Among the formulations, pGE(25) showed the highest modulus, exceeding that of the pE cross-linker homopolymer. Incorporating DMAEMA generally reduced film stiffness relative to epoxide-rich formulations, consistent with the increased chain mobility and viscoelastic character of tertiary amine-containing networks.^50^

In sum, the FTIR, water contact angle, and stiffness results confirm the retention of critical functional groups, i.e., epoxide for bioconjugation and ionizable tertiary amine for modifying the microenvironment of immobilized LacZ.

### 3.2 Morphological Evidence for Immobilization

Atomic force microscopy (AFM) provided direct visualization of LacZ enzyme immobilization on pG films. Figure 2a illustrates the covalent immobilization of LacZ tetramers onto pG via RI and DI, where SC and ST play a key role in the site-directed immobilization of DI. Bare pG displayed a smooth surface (*R*_rms_ = 1.3 ± 0.1 nm) (Figure 2b-i, zoom-in: Figure 2c–i). Following immobilization, distinct granular features appeared, increasing roughness to 3.7 ± 1.3 nm for RI-pG and 4.9 ± 2.7 nm for DI-pG (Figure 2b-ii,iii). BCA quantification assay estimated 48.5 ± 4.6 and 9.1 ± 1.4 LacZ tetramers per 100 nm^2^ for RI and DI, respectively, indicating higher surface coverage for random immobilization. AFM line profiles revealed height distributions of 12–20 nm for RI and 20–36 nm for DI, consistent with the expected size of LacZ tetramers and the additional SpyCatcher-SpyTag complex in DI (Figure 2d). Figure 2 also shows that in both RI-pG and DI-pG, several large features presumed to be clusters of LacZ tetramers (red boxes, Figure 2c-ii,iii) were observed amid vast smaller features, which most likely represent individual LacZ tetramers (blue boxes, Figure 2c-ii,iii). We further verified through Coomassie blue gel and Western Blotting that under the bioconjugation conditions used, LacZ-ST indeed binds to SC, forming LacZ-ST-SC complex with expected molecular weights (Figure S2). In all, these results confirmed successful immobilization of LacZ via both RI and DI methods and their retention on the iCVD-synthesized pG support.

**Figure 2:**
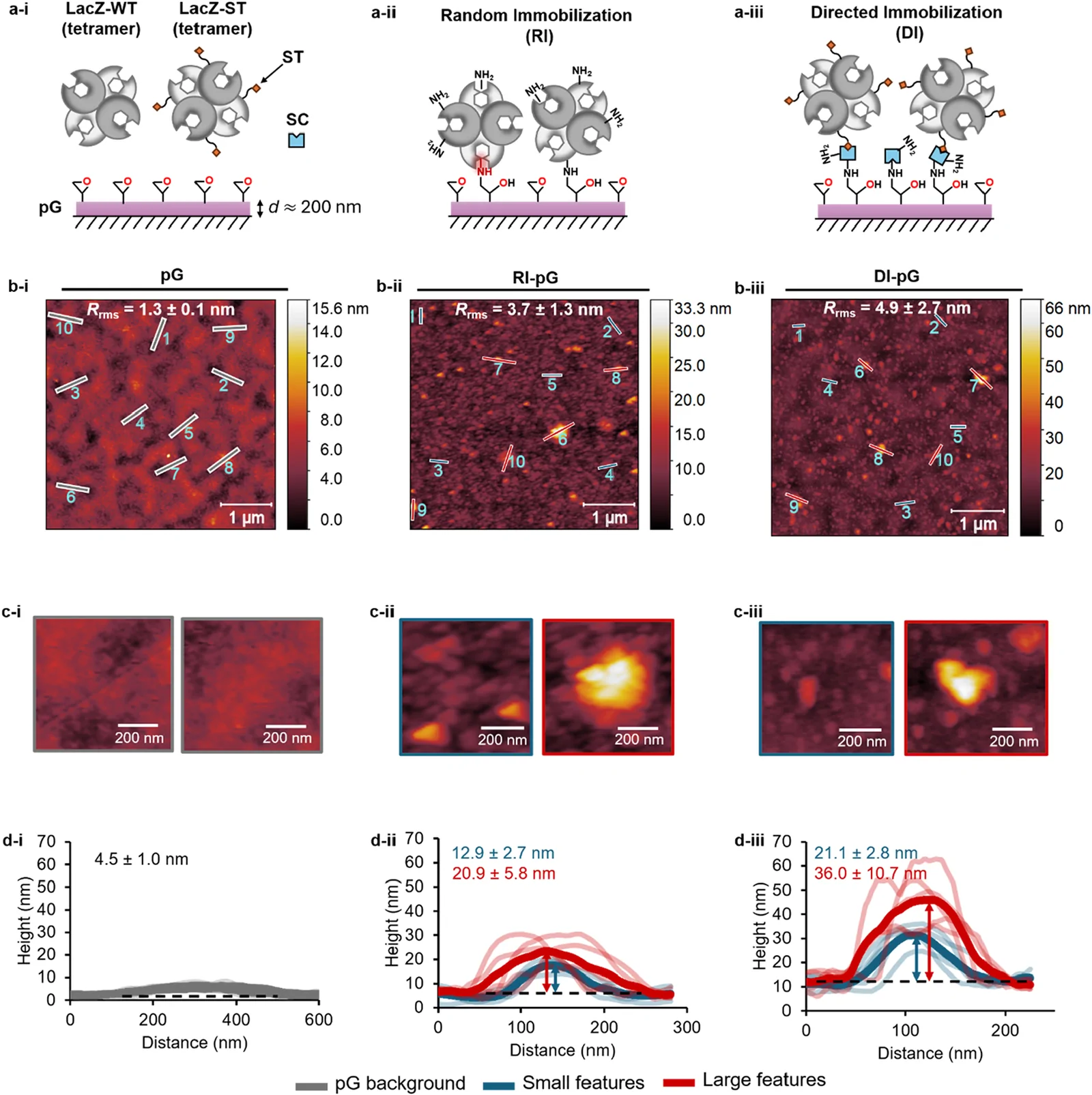
Morphological characterization of immobilized LacZ samples. (a) Schematic illustration of key components involved in immobilization (i), and two immobilization schemes studied here: random immobilization (RI) (ii) and directed immobilization (DI) (iii). Note that amino groups (−NH_2_) are explicitly shown only in LacZ-WT and SC; they are also present on LacZ-ST but are not shown in the illustrations. (b) AFM scan of pG (preconditioned in buffer) (i), pG with randomly immobilized LacZ-WT (RI-pG) (ii), and pG with LacZ-ST via DI (DI-pG) (iii). The lines denote where the line profiles were taken (see panel d). Values of *R*_rms_ and the number of LacZ immobilized per 100 nm^2^ (based on BCA assay) are presented. (c) Representative zoomed-in views corresponding to the large-area scans above. Note that both small (blue box) and large features (red box) are present in both RI (ii) and DI (iii). (d) Line profiles and peak heights (mean ± SD) for pG (i), RI-pG (ii), and DI-pG (iii). Small and large features were color-coded blue and red, respectively.

### 3.3 Chemical Evidence for Immobilization

AFM data provided direct visual confirmation of LacZ immobilization on pG. However, to verify covalent immobilization (e.g., as opposed to physical adsorption), we resorted to signs of epoxide ring opening using FTIR and XPS after immobilization. In principle, amino nitrogen from proteins (LacZ-WT or SpyCatcher) may serve as nucleophiles and attack the epoxy carbon to open the epoxide ring, resulting in the formation of an N–H bond and a β-hydroxyl group (see the reaction scheme in Figure 3a). FTIR spectra in Figure 3b showed, after immobilization, new absorption bands appeared at ∼1640 and ∼1550 cm^–1^ (amide I and II, N–H bending) and broad O–H/N–H stretches between 3100 and 3600 cm^–1^, consistent with formation of β-hydroxyl and amide linkages. Because bound proteins themselves could contribute to these bands (e.g., via peptide bonds) even without epoxide ring opening, and because epoxide ring-opening lacks unique diagnostic peaks, neither FTIR (Figure 3b) nor XPS (C 1s and O 1s; Figures S3 and S4) can conclusively confirm covalent conjugation through epoxide ring opening. A control reaction using glycine was performed to distinguish covalent attachment from physical adsorption. Glycine, the simplest amino acid, contains the same primary amine moiety that LacZ would use for covalent attachment but is much smaller in size, making it easier to rinse away if not truly covalently bonded to the polymer. Thus, glycine can act as a molecular surrogate to verify the occurrence of the epoxide ring-opening reaction under the adopted immobilization conditions without the complications introduced by protein size and conformational heterogeneity. To probe glycine reaction depth relative to the film thickness and confirm covalent immobilization via epoxide-amine ring opening, pG films of different thicknesses (25 and 150 nm) and SG coatings were prepared and exposed to glycine. Incubation produced a clear reduction of the 910 cm^–1^ epoxide peak and appearance of N–H/O–H stretches, confirming amine-epoxide ring-opening reactions at the surface (Figure 3c,d). As pG thickness decreased from 150 to 25 nm, the N–H/O–H band (“1”; Figure 3c) intensified relative to the epoxide band (“4”; Figure 3c), consistent with ring opening occurring predominantly at the film surface. Given that LacZ is larger and less penetrating than glycine, the same surface-localized reaction is expected to dominate during enzyme immobilization, verifying covalent attachment under the described conditions. Moreover, our recent work confirmed that pGD thin films were stable in acidic solution (pH 4), showing no measurable thickness change within experimental error.^43^ No visible delamination or material loss was observed during immobilization, rinsing, or enzyme activity assays, and FTIR analysis confirms retention of characteristic functional groups after treatment.^41^ Together, these results demonstrate that the polymer coatings used in this study are structurally stable during immobilization and catalytic testing.

**Figure 3:**
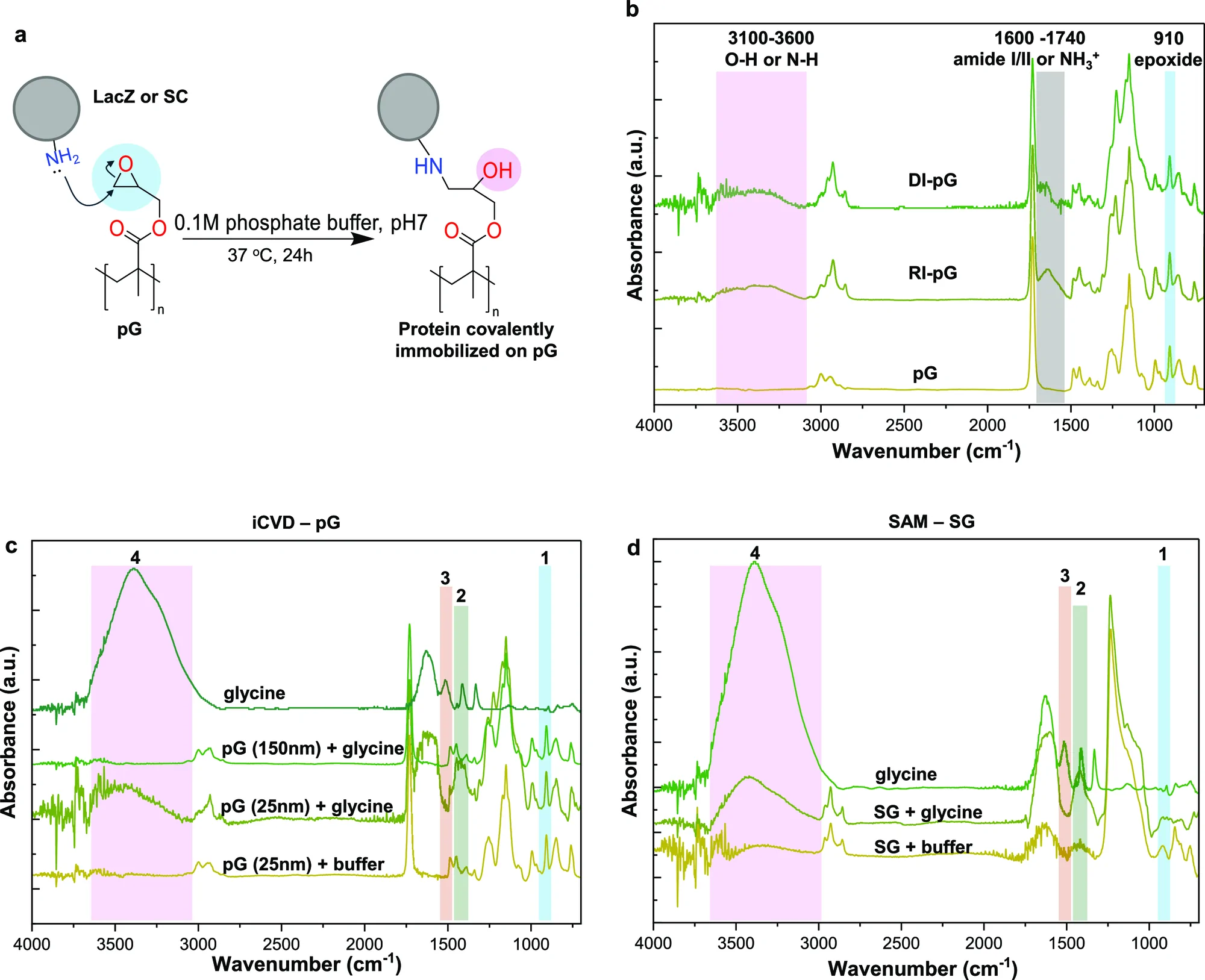
ATR-FTIR evidence for covalent immobilization. (a) Reaction scheme for covalent conjugation of a protein (LacZ or SC) to an iCVD-synthesized polymer support (pG) via the amine-epoxide ring-opening reaction. (b) Spectra of pG (preconditioned in buffer), RI-pG, and DI-pG, after standard rinsing steps. Important bands that signal ring-opening and/or protein immobilization are highlighted, including O–H/N–H stretching (magenta), amide I/II, or NH_3_^+^ (gray), and epoxide ring-deformation vibrations (blue). (c) pG coatings of different thicknesses following glycine blocking. (d) Self-assembled monolayer sample, SG, following glycine blocking. Spectral peak coding: 1:910 cm^–1^, epoxide ring; 2:1413 cm^–1^, COO^–^ symmetric stretch; 3: NH_3_^+^ symmetric bend; 4: overlapped O–H/N–H stretch.

### 3.4 Effect of Site-Directed Immobilization on Enzyme Activity

To investigate the impact of bioconjugation sites on LacZ activity, LacZ was immobilized using RI or DI through specific SC-ST binding. As shown in the schematics of Figure 2a, the main difference between the two bioconjugation methods was that, in the case of DI, SC was first conjugated to immobilization supports via ring opening, followed by specific binding of LacZ-ST to SC; whereas, in the case of RI, any surface-exposed amino groups of LacZ-WT could bond to the epoxide groups. There was no detectable activity under high acidity (pH 4) for either surface-free (LacZ-WT and LacZ-ST) or LacZ immobilized on pG, regardless of bioconjugation methods being RI or DI. Upon random immobilization of wild-type LacZ (LacZ-WT) as in RI-pG, there was a drastic reduction in activity of LacZ, 92% at pH 7 and 93% at pH 8 (*p* < 0.05), in agreement with previous reports.^3,7,8^

In contrast, when LacZ-ST was immobilized via DI (DI-pG), an increase in activity (vs free) was observed at pH 5 (45% increase), pH 6 (57% increase), and pH 8 (20% increase), and activity was retained at pH 7. Comparing RI-pG with DI-pG, there was a significantly higher activity in DI for all pH values evaluated except pH 4, where no activity was detected in either case. There was about a 711% increase in the *v*_0_^*^ of DI-pG relative to RI-pG. This implies that controlling the bioconjugation site of LacZ has a significant impact on improving its activity. This is noteworthy because the immobilization of LacZ has been widely reported to reduce in its activity, rather than a substantial increase.^51–54^

Inclusion of 25 mol % cross-linker EGDMA (E) did not reduce the activity of immobilized LacZ across tested pHs on a per-enzyme basis (*v*_0_^*^), with one exception: DI-pGE at pH 7 (*p* < 0.05) (Figure 4). No enzyme activity was detected at pH 4 for either RI-pGE or DI-pGE (Figure 4), and the optimal pH did not shift, as expected, given the charge neutrality of E. Inclusion of 25 mol % E reduced surface LacZ density and immobilization yield, modestly for RI (−10.9%) and substantially for DI (−53.8%) (Tables S5 and S6), potentially because E replaced epoxide-bearing immobilization sites. Taken together, these cross-linking results suggest that incorporating E improves the rigidity and may improve the stability of iCVD supports while generally preserving per-enzyme LacZ activity. However, EGDMA lowers the immobilization yield and may therefore reduce the total activity, depending on whether the retained per-enzyme activity offsets the reduced enzyme loading.

**Figure 4:**
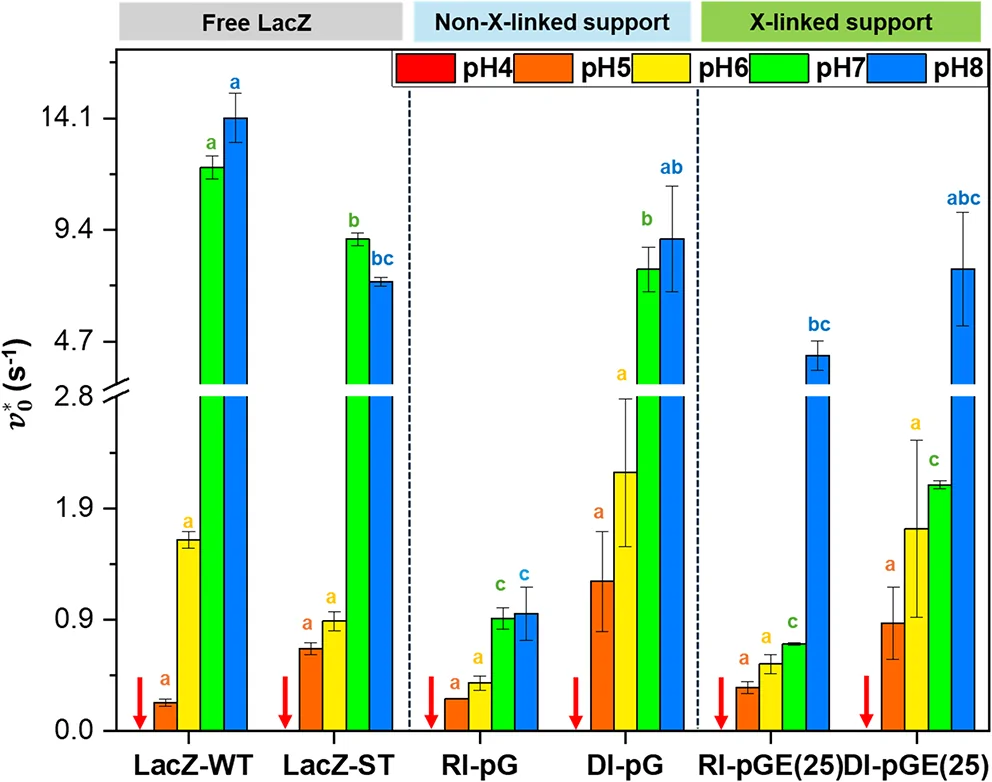
Normalized initial rate (*v*_0_^*^) of LacZ in free-floating and immobilized forms across a pH range of 4 to 8. LacZ-WT and LacZ-ST represent the free-floating enzymes used in RI and DI, respectively. Nonoverlapping letters of the same color (matching the pH condition) denote significant differences among samples measured at that pH (*p* < 0.05). Red arrows indicate no detectable enzyme activity at pH 4. Error bars represent standard errors (*N* = 3).

Two caveats affect this interpretation. First, during DI, glycine was introduced following initial SC immobilization to block residual unreacted epoxide groups, thus minimizing the likelihood of LacZ-ST reacting nonspecifically through exposed amines. Ineffective blocking could lead to unwanted amine-epoxide interactions competing with the intended DI step–specific LacZ-ST immobilization via ST-SC binding. Although the FTIR data indicated extensive glycine-mediated epoxide ring opening (Figure 3c,d), the spectra alone could not establish that all residual epoxide groups had been blocked. Therefore, we further investigated the efficiency of blocking unreacted epoxide with glycine^55^ (1.0 M in pH 7 phosphate buffer, 37 °C, 24 h). We compared the extent of random LacZ-ST immobilization on pG following standard incubation conditions (pH 7, 2 h, 25 °C, 30 rpm) with or without the prior glycine-blocking step. Our results indicate that glycine blocking did not completely prohibit LacZ-ST immobilization via RI but substantially reduced (−40%) such nondirected immobilization (Figure S5). This finding suggests that the DI protocol results in predominantly SC-ST-mediated immobilization with a residual nonspecific component.

Second, immobilization via RI consistently resulted in a higher immobilization yield (IY) and surface density (Γ) on pG compared to DI (Figure 5a). This higher surface density under RI conditions could decrease the normalized initial rate (*v*_0_^*^) due to molecular crowding,^56^ potentially causing steric hindrance and limiting substrate accessibility to the LacZ active sites.^57,58^ Indeed, as shown in Figure 5a, reducing the surface density of RI-pG from approximately 45 LacZ tetramers (LacZ_4_) per 100 nm^2^ under the high-loading condition (“H”), used in Figure 4 and subsequent experiments, to approximately 15 LacZ_4_per 100 nm^2^ under the low-loading condition (“L”) increased *v*_0_^*^. This trend is consistent with previous reports on randomly immobilized xylanases^59^ and LacZ.^60^ Interestingly, the trend of *v*_0_^*^ relative to surface density was reversed for DI-pG samples: the higher-density DI-pG­(H) exhibited approximately 250% greater *v*_0_^*^ compared to DI-pG­(L) (Figure 5b). When matched for surface density (see purple columns in Figure 5a, Γ ≈ 50 LacZ_4_ counts per 100 nm^2^), *v*_0_^*^ of DI-pG­(H) remained more than 10-fold higher than that of RI-pG­(H) (see maroon columns in Figure 5b). This result indicates that DI’s improved activity was not solely due to reduced crowding effects, but rather due to inherently superior enzyme activity resulting from the DI approach. Therefore, surface-density-dependent LacZ activity differed between RI and DI under the tested conditions, consistent with prior work showing that crowding effects depend strongly on surface orientation of immobilized enzymes.^61^

**Figure 5:**
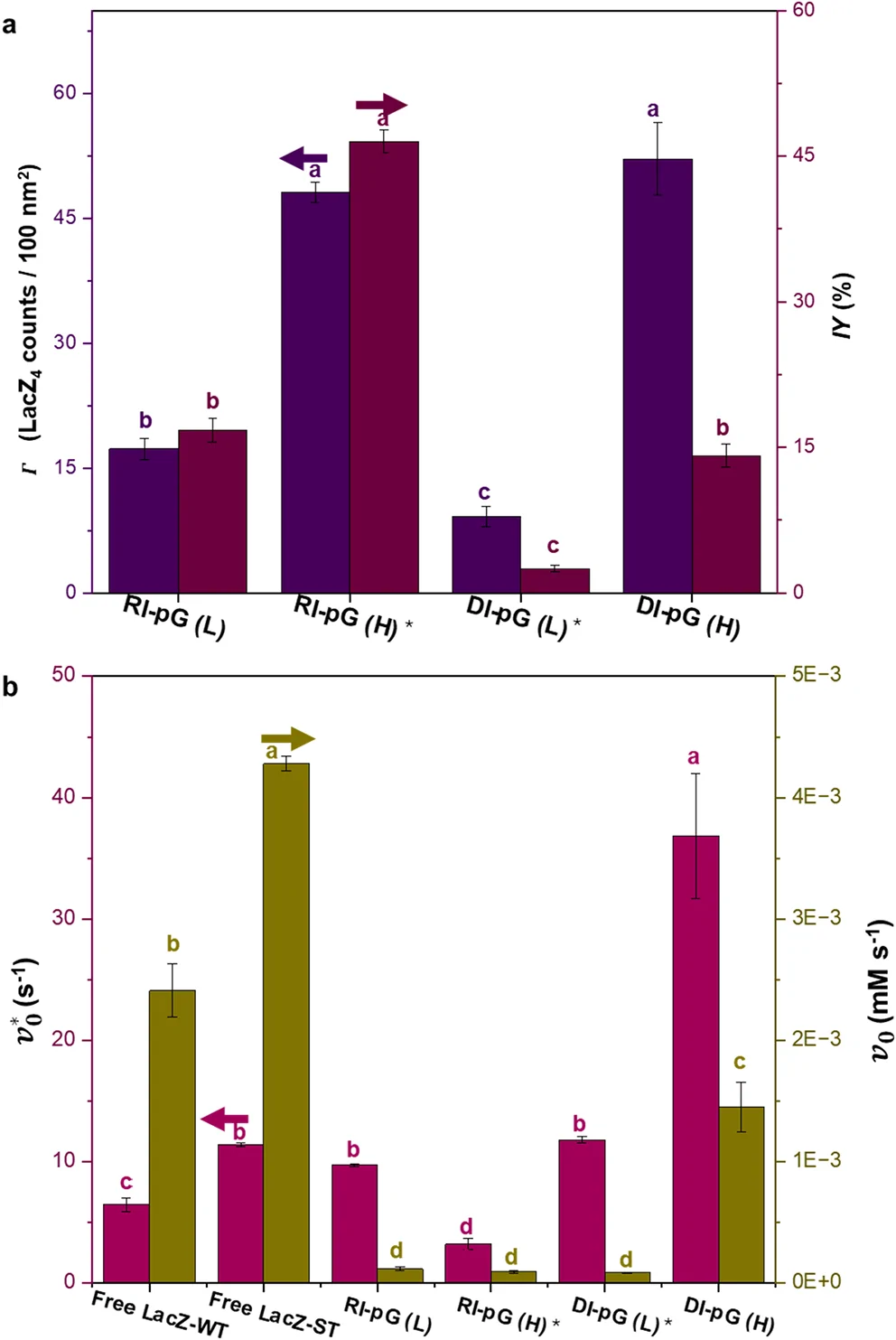
Effect of surface density of LacZ tetramers (LacZ_4_) on enzyme activity. (a) Surface density (Γ) and immobilization yield (*IY*) under high- (“H”) and low-loading (“L”) conditions for LacZ_4_ immobilized on pG via RI or DI. (b) Initial rate (*v*_0_) and normalized initial rate (*v*_0_^*^, normalized by concentration of active sites, i.e., LacZ monomer concentration) measured for LacZ in free-floating form and immobilized on pG under high- and low-loading conditions via RI or DI. Immobilization protocols for samples labeled with an asterisk (*), i.e., RI-pG (H) and DI-pG (L), were adopted for experiments in the rest of this work. Error bars represent standard errors (*N* = 3).

### 3.5 Effect of Polycationic Moieties on Enzyme Activity

To test the hypothesis that the protonatable tertiary amines of DMAEMA (“D”), occurring at pH less than p*K*_a_ (8.4) (see Figure 6a), can buffer the local microenvironment of the immobilized LacZ and enable biocatalysis at bulk pH 4, we synthesized pGD(25), pGD(65), and pGDE (37%D, 14%E) using iCVD. The molar compositions of these copolymers were confirmed by FTIR (see Figure 1b and Table S3 for detailed). LacZ activity, shown as *v*_0_^*^ in Figure 6b,c, revealed a strong correlation between the presence of polycationic moieties and preservation of activity under high acidity. Specifically, for “Polycationic −” samples, regardless of RI or DI or free enzymes (LacZ-WT or LacZ-ST), no activity was observed at pH 4 (denoted by red arrows). In contrast, whenever DMAEMA was present in the copolymers (“Polycationic + ”), activity was observed consistently at bulk pH 4, with varying degrees. Comparing bioconjugation methods, DI invariably exceeded RI in observed activity at pH 4 for all polymer supports tested, including the polycationic types, i.e., pGD(25), pGD(65), and pGDE (Figure 6b,c). This result aligns with the observed DI-mediated enhancement in LacZ activity, likely arising from the combined effects of improved enzyme activity via site-specific DI and reduced molecular crowding compared to RI, as previously discussed (Figure 5). The lack of activity for all DIs under “Polycationic–” conditions at pH 4 indicates that the proteinaceous SpyCatcher–SpyTag binding layer introduced during DI does not buffer against acid stress but instead primarily reduces conformational strain by controlling bioconjugation sites and enzyme orientation.

**Figure 6:**
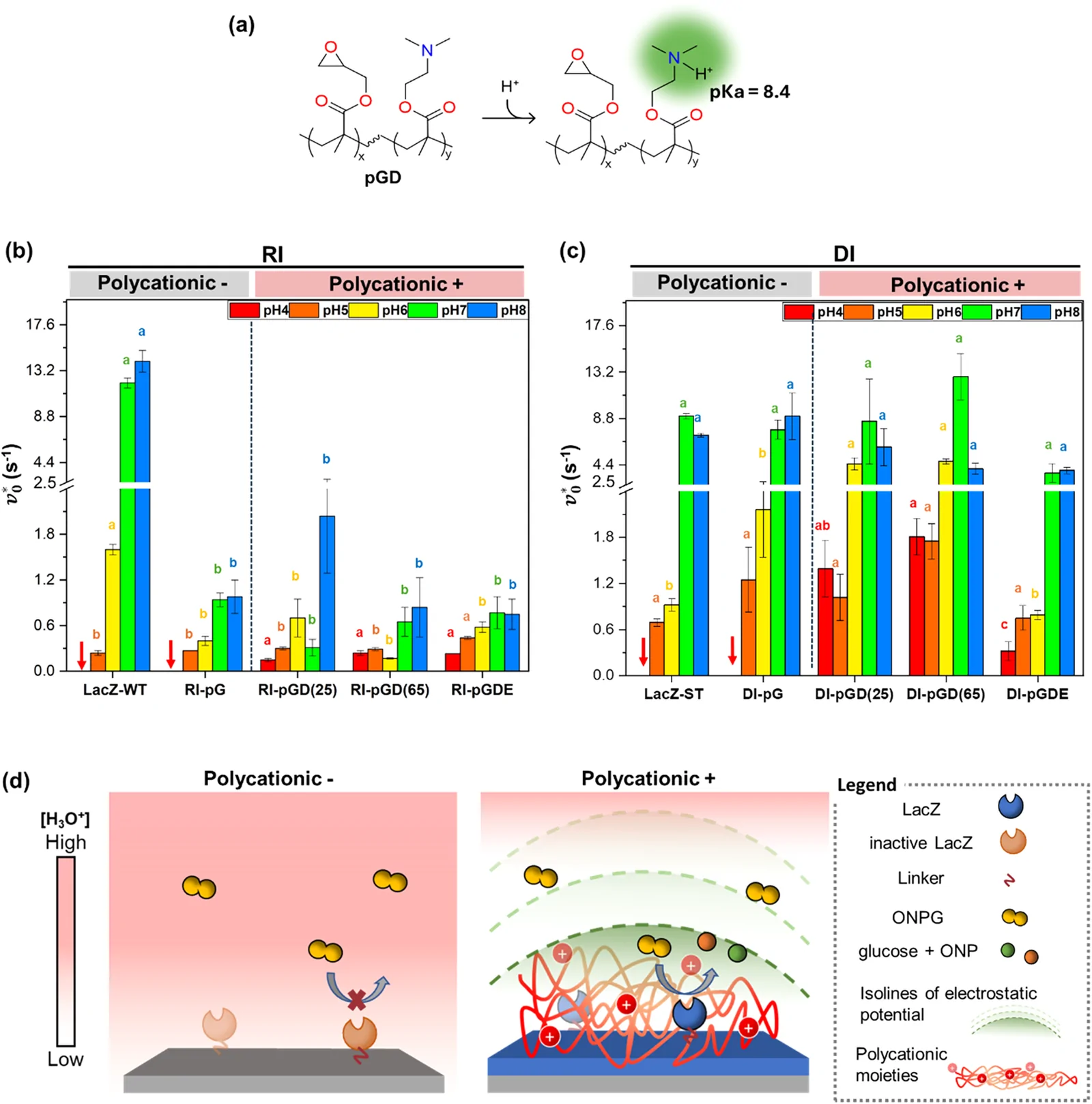
Effect of cationic moieties on LacZ activity. (a) Reaction scheme illustrating protonation of the tertiary amine moiety of DMAEMA at pH lower than its p*K*_a_ (8.4). (b, c) Normalized initial rate (*v*_0_^*^) of LacZ across pH 4–8: (b) LacZ-WT and RI samples, and (c) LacZ-ST and DI samples. Nonoverlapping letters of the same color (matching the pH condition) denote significant differences among samples measured at that pH (*p* < 0.05). Red arrows denote no detectable enzyme activity at pH 4. Error bars represent standard errors (*N* = 3). (d) Proposed mechanism by which polycationic polymer supports generate protective electrostatic shielding, repelling hydronium ions and thus preserving LacZ activity under highly acidic conditions.

Comparing polymers with various proportions of polycations: in the case of DI (Figure 6c), pGD(65) resulted in a LacZ activity of 1.8 ± 0.2 s^–1^ at bulk pH 4, 82.2 and 29.6% higher than pGDE and pGD(25), respectively. This was expected considering the p*K*_a_ of the tertiary amine group in DMAEMA being 8.4,^62–64^ and at bulk pH 4, nearly all tertiary amines are predicted to be positively charged based on the Henderson–Hasselbalch equation.^43^ These positively charged, protonated tertiary ammonium group in turn would electrostatically push the protons and hydronium ions (H_3_O^+^) away from the polymer surface. This electrostatic repulsion created a protective shield for the immobilized LacZ (Figure 6d). Thus, local pH increase is expected to be greatest for pGD(65).^67^ Additionally, for DI-pGD(65) and DI-pGD(25), a downward shift in optimal pH was observed relative to DI-pG, consistent with earlier studies on enzymes immobilized on positively charged supports, such as polyethylenimine^65^ and chitosan.^66^ Also, we noticed encouraging retention, even improvement in certain cases of LacZ activity by using the site-specific DI approach, under more neutral pHs (Figure 6c). Particularly, comparing DI-pGD(65) with its free LacZ control, LacZ-ST, we observed 41.7 and 410.9% activity increase at pH 6 and pH 7, respectively. In contrast, when RI was used for immobilization instead of DI, a substantial reduction in activity was observed with RI-pGD(65) relative to its free LacZ control, LacZ-WT (Figure 6b), consistent with earlier results for RI (Figure 5). Also, it is noteworthy that the prominent, D-content-dependent pH-buffering effect observed with DI (Figure 6c) appeared minimally under RI conditions (RI-pGD(25), RI-pGD(65), and RI-pGDE; Figure 6b).

It is widely reported that the activity of many enzymes, including LacZ, often lowers upon immobilization due to conformational strains at the active site or reduced substrate accessibility, posing a major limitation for various enzyme immobilization technologies. The acid sensitivity of LacZ arises from both protonation of catalytic residues (Glu461 and Glu537) and partial structural destabilization, as confirmed by a gradual loss in α-helical content with decreasing pH (Figure S8). Thus, the polycationic supports likely protect immobilized enzymes by preserving a favorable local pH microenvironment, thereby mitigating detrimental protonation at the catalytic sites and maintaining the conformation required for normal enzymatic function.

A limitation of the present ONPG-based activity assay is that the molar absorptivity of ONP at 420 nm decreases under acidic conditions (Figure S7). Because ONP ionization and LacZ activity both vary with pH, the uncorrected 420 nm absorbance signal likely underestimates the absolute amount of ONP formed under acidic conditions (because protonated ONP has weaker 420 nm absorbance) and therefore may underestimate the protective effect of the polycationic supports. This limitation affects absolute activity estimates and direct comparisons across pH; however, comparisons among samples assayed under the same pH condition remain internally valid throughout this study. Future studies may either apply pH-specific ONP calibration factors or monitor ONP formation near its isosbestic wavelength ∼373 nm,^67^ where the charged and uncharged forms of ONP have equivalent molar absorptivity and the absorbance is therefore less sensitive to ONP ionization state.

Overall, the results presented here demonstrate that site-directed bioconjugation on the surface of the support, coupled with polymer charge engineering, can preserve or even enhance immobilized LacZ activity relative to their free counterparts, at both optimal (pH 7) and suboptimal (pH 4) conditions.

### 3.6 SAM vs iCVD Coatings as Enzyme Immobilization Supports

To compare the performance of SAM and iCVD coatings, we synthesized SAMs containing either solely an epoxide end group (SG) or a combination of epoxide (75 mol %) and tertiary amine (25 mol %) end groups, SGD(25), nominally matching the functional-group composition of iCVD coatings pG and pGD(25), respectively. Covalent immobilization of LacZ onto these two SAM supports via RI and DI was previously confirmed (Figure 3d). As illustrated in Figure 7a,b, the SAM and iCVD supports were designed with nominally matched principal functional groups and compositions but differed primarily in backbone structure and coating thickness. The SAM coatings were less than 7 nm thick, as measured by ellipsometry, and were expected to be relatively uniform across the well bottoms and side walls. In contrast, the iCVD films were approximately 200 nm thick at the well bottoms and 50 nm thick along the upper side walls. Below, we examine how these differences in the coating thickness influence enzyme loading and activity.

**Figure 7:**
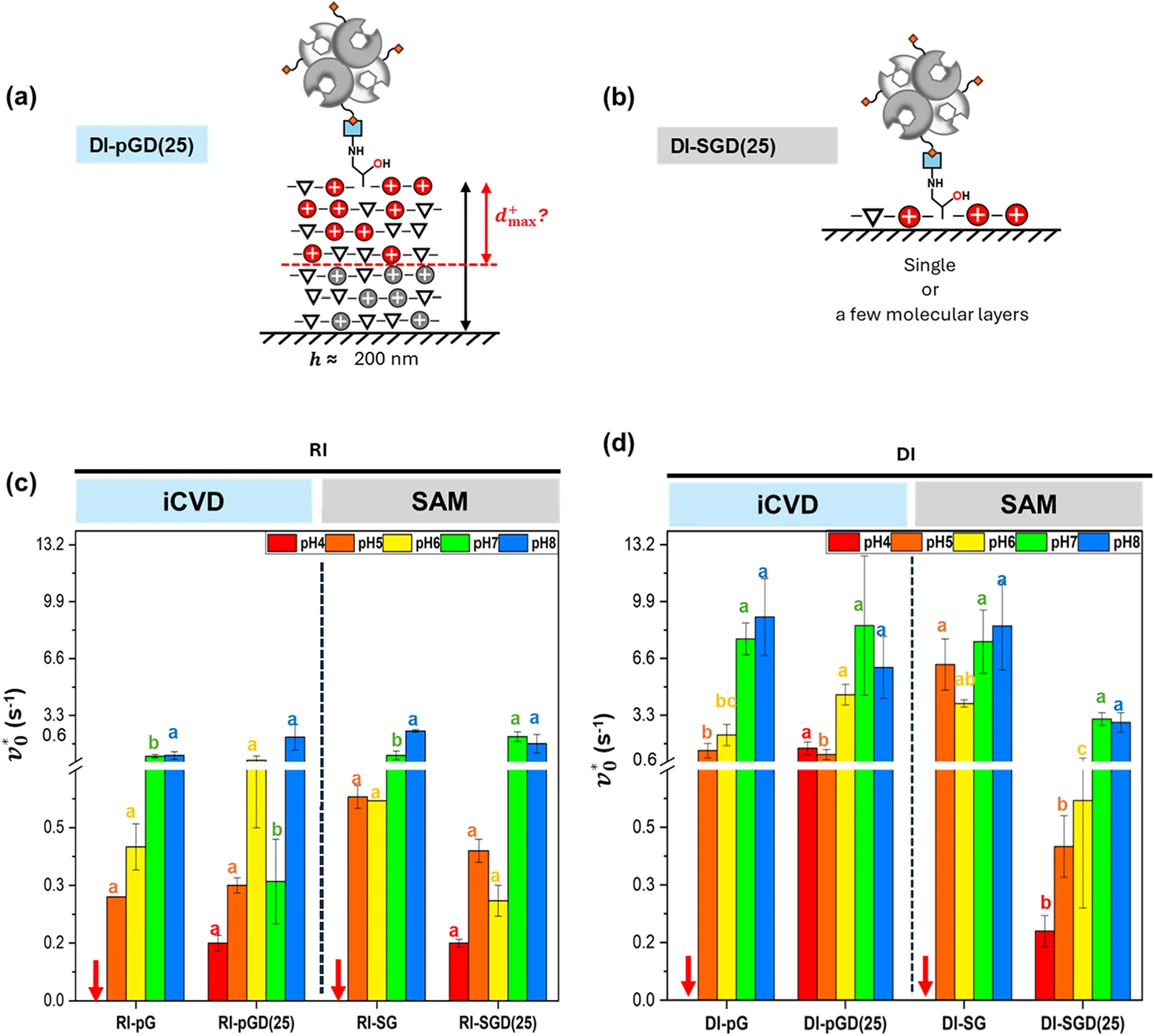
Comparison of LacZ activity on iCVD and SAM supports with matched surface chemistry. (a, b) Schematic illustrations of DI on two polycationic immobilization supports: (a) iCVD-synthesized pGD(25), where *h* and *d*_max_^+^ denote film thickness and protonated layer thickness, respectively; and (b) SAM-synthesized, SGD(25) monolayer. (c, d) Normalized initial rate (*v*_0_^*^) of LacZ across pH 4–8 for: (c) RI samples (LacZ-WT), and (d) DI samples (LacZ-ST). Nonoverlapping letters of the same color (matching the pH condition) denote significant differences among samples measured at that pH (*p* < 0.05). Red arrows denote no detectable enzyme activity at pH 4. Error bars represent standard errors (*N* = 3).

First, regarding enzyme loading, the amounts of LacZ immobilized on SG and SGD(25) were comparable to those on pG and pGD(25), respectively, for both RI and DI (Tables S5 and S6). The immobilization method (RI vs DI) had a greater influence on enzyme loading than the support thickness (iCVD vs SAM). Specifically, RI-SG and RI-SGD(25) yielded 8.5-fold and 5.5-fold higher enzyme loadings than DI-SG and DI-SGD(25), respectively (Tables S5 and S6), consistent with the higher RI loading observed for pG under the selected immobilization conditions (Figure 5a). Thus, the reduced thickness of SAM coatings did not result in lower enzyme loading relative to their iCVD counterparts, consistent with earlier observations that immobilization predominantly occurred on the support surfaces rather than within their bulk. Second, we observed a notable interplay among three factors: (i) support thickness, (ii) polycationic chemistry, and (iii) immobilization method. Specifically, at pH 4, the thicker DI-pGD(25) yielded 8-fold higher LacZ activity than its monolayer counterpart, DI-SGD(25) (Figure 7d), likely because the 200 nm iCVD coating presents far more protonatable tertiary amine moieties per unit area, thus greater protection of LacZ active sites from acid stress, than the monolayer (Figure 7a,b). In contrast, RI showed little thickness-dependent improvement at pH 4 or pH 5 for RI samples (RI-pGD(25) vs RI-SGD(25); Figure 7c). DI increased activity by ∼10-fold relative to RI on pGD(25) at pH 4 (Figure 7d,c), consistent with DI’s superior performance in preserving enzyme activity in general.

A potential confounding factor is well geometry. As noted in Section 2.3, iCVD supports exhibit a thickness gradient within the well (e.g., ∼200 nm at the bottom vs ∼50 nm at the upper sidewall) due to temperature variations. Our recent work shows that this gradient has minimal impact on immobilization yield and enzyme surface density but may affect acid protection by the polycationic pGD coating.^41^ Specifically, LacZ acid resistance increased with pGD thickness, plateauing at ∼250 nm due to limits in proton penetration within the polycationic films.^41^ Accordingly, under acid stress, LacZ immobilized on the sidewall regions with thinner polycationic supports may be less protected than that on the well bottom. This spatial effect is likely negligible for SAMs, given their uniform monolayer thickness.

### 3.7 Kinetic and Mechanistic Interpretation

To investigate the mechanisms of activity change, we extracted Michaelis–Menten kinetic parameters through regression from *v*_0_^*^ vs [ONPG] plots (range: 0 to 16.6 mM) for all of the supports tested under pH 4 and 7 (Tables S7–S10). Under pH 7, DI samples exhibited higher catalytic efficiencies (*k*_cat_/*K*_m_) relative to RI for all iCVD supports, i.e., pG, pGE, pGD(25), pGD(65), and pGDE, ranging from +69.4% (pGD(25)) to +3894.4% (pGD(65)), consistent with earlier *v*_0_^*^ data (Figures 4 and 6); however, under pH 4, *k*_cat_/*K*_m_ of DI showed a mixed outcome relative to that of RI: it decreased for pGD(25) (−14.0%) and pGDE (−53.6%) but increased for pGD(65) (+44.1%).

DI did not consistently reduce *K*_m_ relative to RI, suggesting that the improved performance from DI primarily resulted from maintaining a higher turnover number (*k*_cat_) at the enzyme’s active site, likely due to reduced conformational strain postimmobilization, rather than from improved ONPG binding affinity. This phenomenon is exemplified by supports composed solely of epoxide groups, comparing RI-pG with DI-pG, *k*_cat_ increased from 1.34 to 9.32 s^–1^ (+594%), while *K*_m_ increased from 1.82 mM to 3.96 mM (+118%) (Figure S6, Tables S7 and S9). As shown in Figure 8, the pattern that DI (solid symbols) increases both *k*_cat_ and *K*_m_ relative to RI (hollow symbols) also holds true for most polycationic supports, with the exception of pGD(25) (black) (Figure 8). This observation seems to contrast prior reports in which DI primarily improved apparent binding affinity by reducing obstruction of the active site.^42,68^ One explanation for this discrepancy is that LacZ is immobilized as a tetramer (LacZ_4_), whereas many prior studies focused on monomeric enzymes. As such, the likelihood that unfavorable LacZ_4_-support interactions simultaneously block ONPG access at all four active sites is much lower than that for a monomeric enzyme with only one active site.

**Figure 8:**
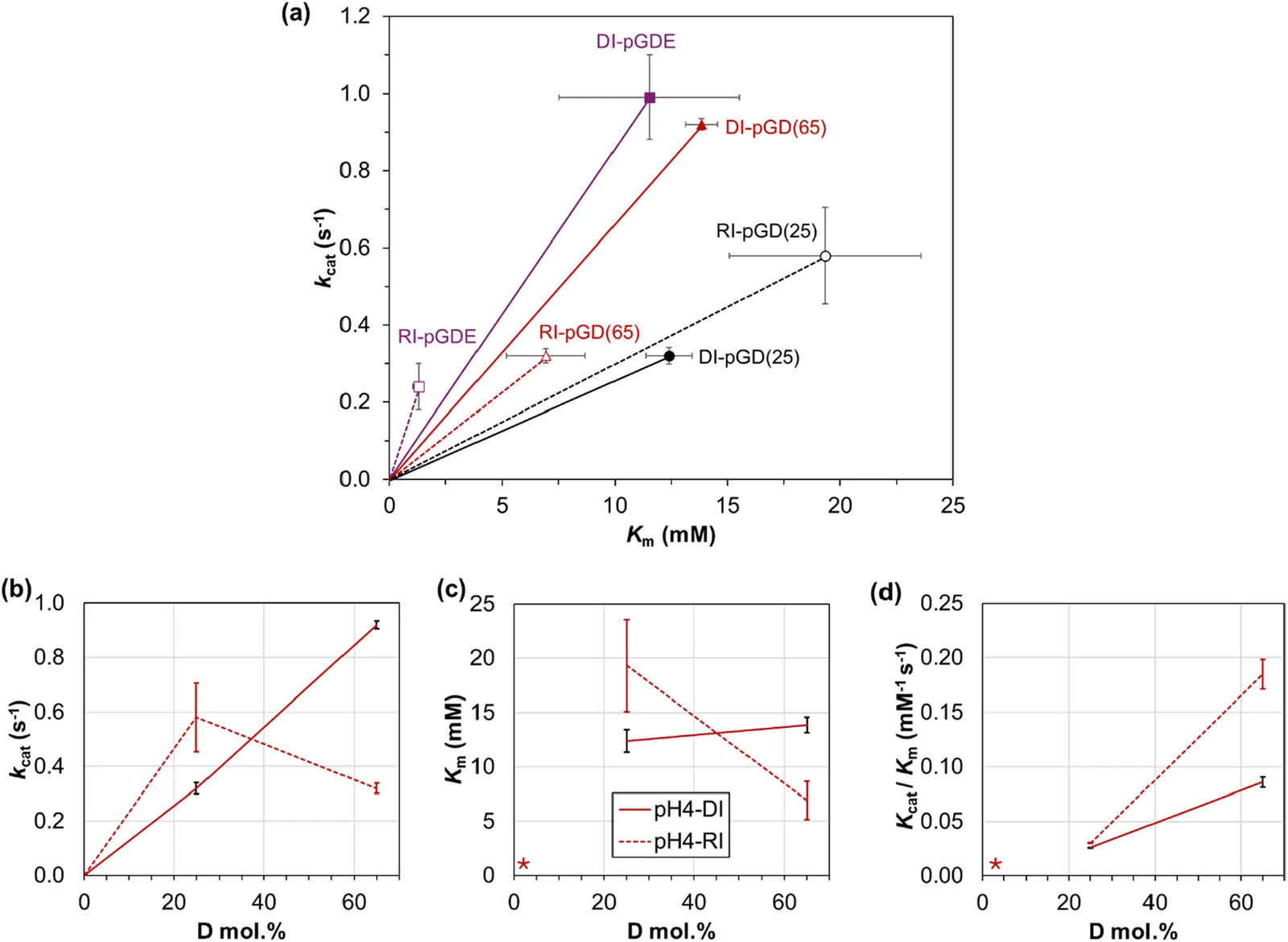
Kinetic effect of polycationic content, cross-linking, and immobilization method. (a) Comparison of Michaelis–Menten kinetic parameters for samples exhibiting detectable enzyme activity at pH 4, with the slopes representing catalytic efficiency (*k*_cat_/*K*_m_). (b–d) *k*_cat_, *K*_m_, and *k*_cat_/*K*_m_ as functions of polycationic contents in iCVD supports: pG, pGD(25), and pGD(65) represent 0, 25, and 65 mol % of D, respectively. Red asterisks in parts (c) and (d) denote values not obtainable, due to LacZ activity not detected at pH 4. Error bars represent standard errors (*N* = 3).

For SAM supports, switching the immobilization method from RI to DI on SG and SGD(25) surprisingly did not replicate the DI-mediated improvement in LacZ catalytic efficiency; instead, it reduced catalytic efficiency under pH 7 for SG (−85.4%) and SGD(25) (−13.5%). This switch to DI also resulted in an inconsistent *K*_m_ increase for SG (+551.9%) and SGD(25) (+196.6%) under pH 7, suggesting lower ONPG-LacZ binding affinity. Under pH 4, DI increased the *k*_cat_ of SGD(25) drastically relative to RI (+1123.1%). These results from SAM supports suggest a similar trend with iCVD supports that DI tends to increase both *K*_m_ and *k*_cat_ of immobilized LacZ_4_ relative to RI.

When evaluating pH effects, both RI and DI on polycationic supports (iCVD- and SAM-based) retained detectable activity at pH 4 (Tables S8 and S10). Switching from iCVD pGD(25) to SAM SGD(25) reduced *k*_cat_ by about 77.6% at pH 4 under RI (Table S8). Figure 8a compares simultaneously the kinetic parameters, *K*_m_, *k*_cat_, and *k*_cat_/*K*_m_ (the slope) obtained on iCVD supports at pH 4 (only those with detectable activity were displayed). Polycationic chemistry proved essential in preserving immobilized LacZ activity at pH 4, as none of the free-floating LacZ exhibited detectable activity under these conditions, consistent with earlier results (Figures 6 and 7). Intriguingly, the inclusion of cross-linker E (see samples denoted by purple symbols and lines in Figure 8a) in iCVD supports appeared to enhance *k*_cat_/*K*_m_, as reflected by the steeper slopes, primarily by reducing *K*_m_, suggesting that reduced chain mobility in the local environment improves apparent ONPG-LacZ binding affinity. Evaluation of polycationic content effects on kinetic parameters revealed that at pH 4, both *k*_cat_ and *k*_cat_/*K*_m_ increased between 25 and 65 mol % D (Figure 8b,d). For RI, *K*_m_ decreased with increasing D mol % in pGD, but no clear change was observed for DI (Figure 8c).

Overall, at pH 4, DI-pGDE and DI-pGD(65) formed the highest-performing group among the iCVD supports under DI, with no significant differences in *k*_cat_or *k*_cat_/*K*_m_. DI-pGDE achieved *k*_cat_ = 0.99 ± 0.11 s^–1^ and *k*_cat_/*K*_m_ = 0.09 ± 0.02 mM^–1^ s^–1^, whereas DI-pGD(65) achieved *k*_cat_ = 0.92 ± 0.01 s^–1^ and *k*_cat_/*K*_m_ = 0.07 ± 0.00 mM^–1^ s^–1^(Figure 8a). Across all samples, RI-pGDE exhibited the highest catalytic efficiency, *k*_cat_/*K*_m_ = 0.18 ± 0.01 mM^–1^ s^–1^, despite its relatively low *k*_cat_ of 0.24 ± 0.06 s^–1^. In contrast, DI-SGD(25) exhibited the highest *k*_cat_, 1.59 ± 0.15 s^–1^, with *k*_cat_/*K*_m_ = 0.10 ± 0.01 mM^–1^ s^–1^. At pH 7, DI-pG outperformed all immobilized samples by exhibiting the highest *k*_cat_ (9.32 ± 1.18 s^–1^), even higher than that of free-floating LacZ-ST (*k*_cat_ = 5.18 ± 0.13 s^–1^) while maintaining a competitive *k*_cat_/*K*_m_ (2.36 ± 0.02 mM^–1^s^–1^) (Figure S6, Table S9). In this study, kinetic assays were performed without supplemental metal-ion cofactors. Optimizing cofactor (e.g., Mg^2+^ for *E. coli* LacZ) concentrations for the immobilized system may further increase *k*_cat_ and *k*_cat_/*K*_m_.

Notably, optimal *k*_cat_/*K*_m_ and *k*_cat_ did not always coincide within samples. For instance, at pH 4, RI-pGDE exhibited the highest *k*_cat_/*K*_m_ (0.18 ± 0.01 mM^–1^s^–1^) but the lowest *k*_cat_ (0.24 ± 0.06 s^–1^) (Figure 8a). Such divergence motivates a brief discussion of whether *k*_cat_/*K*_m_ or *k*_cat_ should serve as the primary performance metric for identifying the optimal coengineering design. For single-substrate Michaelis–Menten behavior (not considering the effect of substrate inhibition), the initial rate is defined by eq 2

2 $$v_{0}=\frac{k_{\mathrm{cat}}{\left[E\right]}_{T}\left[S\right]}{K_{m}+\left[S\right]}$$

where *v*_0_ is the initial rate, [E]_T_ is the total enzyme concentration, and [S] is the substrate concentration.

When the local substrate concentration at the immobilized enzyme satisfies [S]_local_ ≫ *K*_m_ (or ≳10 *K*_m_) and transport limitations are negligible, the observed initial rate *v*_0_ approaches *V*_max_ ≈ *k*_cat_[E]_T_, so improving *k*_cat_ (and active enzyme loading) is the most direct route to higher productivity. When [S]_local_ is comparable or smaller than *K*_m_ (e.g., due to limited bulk supply or unfavorable diffusion/partitioning), performance becomes sensitive to *k*_cat_/*K*_m_, which governs the low-substrate regime. As a model system for lactose, ONPG is less soluble in aqueous buffers near-ambient temperatures, typically in the double-digit millimolar range in common aqueous buffers, and hence both *k*_cat_/*K*_m_ and *k*_cat_ should be considered. By comparison, lactose is soluble at ∼500–700 mM,^69,70^ which may place reactions in the [S]_local_ ≳10 *K*_m_ regime where *k*_cat_, rather than *k*_cat_/*K*_m_, dominates.

## 4 Conclusion

This work establishes a materials-based framework to enhance enzyme performance under acid stress by coengineering the immobilization strategy and local microenvironment. Using initiated chemical vapor deposition (iCVD), an all-dry, vapor-phase support fabrication process, we synthesized modular immobilization supports containing epoxide groups (GMA) for covalent immobilization, ionizable tertiary amines (DMAEMA) for modifying the local electrostatic environment, and cross-linkers (EGDMA) for adjusting film rigidity. Morphological and chemical characterization supported successful enzyme immobilization through random immobilization (RI) and a predominantly SpyCatcher/SpyTag-mediated directed immobilization (DI) protocol, although residual nonspecific attachment may occur in DI samples.

The effects of DI on enzymatic kinetics were pH- and support-dependent. For iCVD supports at pH 7, DI generally increased catalytic efficiency relative to RI, primarily by maintaining a higher turnover number, *k*_cat_, rather than consistently decreasing the Michaelis constant, *K*_m_. However, DI did not universally improve *k*_cat_/*K*_m_, particularly for pGD(25) and pGDE at pH 4 and for the SAM supports SG and SGD(25) at pH 7. These findings indicate that the benefits of controlling the immobilization pathway depend on support architecture and chemistry and may reflect the combined effects of enzyme orientation, conformational constraints, and orientation-dependent crowding.

At pH 4, ionizable tertiary amine groups were essential for preserving detectable LacZ activity under both the RI and DI. Within the non-cross-linked DI-pGD series, *k*_cat_ and *k*_cat_/*K*_m_ increased between 25 and 65 mol % DMAEMA. This result is consistent with electrostatic shielding that may mitigate detrimental protonation of catalytic residues, although the local pH and proton distribution were not measured directly in this study. Additionally, when the nominal polycationic content was held constant at 25 mol %, the iCVD film pGD(25), approximately 200 nm thick at the well bottom, exhibited an approximately 8-fold higher normalized initial rate than the corresponding SAM support, SGD(25), at pH 4. This enhancement was detected under DI but not RI, further demonstrating that the influence of the support architecture depends on the immobilization strategy.

Overall, the optimal support design depends on the selected performance metric. At pH 4, DI-pGDE and DI-pGD(65) formed the highest-performing group among DI-iCVD supports based on *k*_cat_ and *k*_cat_/*K*_m_, whereas RI-pGDE exhibited the highest *k*_cat_/*K*_m_ across all samples but had a comparatively low *k*_cat_. Therefore, a formulation optimized for catalytic efficiency may not maximize turnover under substrate-saturating conditions, highlighting the need to consider the anticipated substrate concentration during conversion. Collectively, these results demonstrate that the immobilization strategy and support microenvironment jointly influence LacZ kinetics and that coengineering both can improve selected measures of enzyme performance under acidic processing conditions.

## Supplementary Material



## Notes

Nanoindentation methodology; surface wettability and elastic modulus; confirmation of LacZ-SpyTag/SpyCatcher bioconjugation; XPS spectra; glycine-blocking evaluation; Michaelis–Menten kinetic comparisons; pH-dependent *o*-nitrophenol calibration curves; circular dichroism spectra; iCVD process parameters, pressure saturation ratios, deposition rates, and copolymer compositions; primer and SpyCatcher gene-block information; random and directed immobilization efficiencies; kinetic parameters at pH 4 and 7; and polymer-film thicknesses and nanoindentation penetration depths (Figures S1–S8 and Tables S1–S11) (PDF)
