# Supplementary material for: Coengineering Vapor-Deposited Polycationic Microenvironments and Immobilization Strategy to Enhance β‑Galactosidase Kinetics at Acidic pH
Source: ACS Omega. 2026 Aug 17;11(34):50892–907. doi: 10.1021/acsomega.6c02352 (PMC13624946; doi:10.1021/acsomega.6c02352)
Supplement: Supplementary file 1 [file ao6c02352_si_001.pdf]

Supporting Information

Co-Engineering Vapor-Deposited Polycationic Microenvironments and Immobilization Strategy to Enhance β-Galactosidase Kinetics at Acidic pH

Felicia Fianu[1], Junxing Chen[2], Babak Faraji Gougerdchi[1], Huida Duan[1], Haibo Huang[1], Rong Yang[3], Wei Sun[2*], Yifan Cheng[1*]

1. Virginia Polytechnic Institute and State University, Department of Food Science and Technology, Blacksburg, VA 24061
2. Virginia Polytechnic Institute and State University, Department of Biochemistry, Blacksburg, VA 24061
3. Cornell University, Smith School of Chemical and Biomolecular Engineering, Ithaca, NY 14853

*Correspondence:

Prof. Yifan Cheng, email: yifancheng@vt.edu

Prof. Wei Sun, email: sunwei@vt.edu

Supporting Information

Methodology of nanoindentation

Nanoindentation measurements were performed using a 50 nm radius Berkovich diamond tip with a maximum applied load of 150 µN to evaluate the elastic properties of the polymer thin films. The penetration depth of the tip as well as the thickness of each polymer is tabulated in **table S11**. The elastic modulus was determined from the unloading segment of the load-displacement curve using the Oliver-Pharr method[1,2], which calculates the reduced modulus from the contact stiffness and projected contact area. Young's modulus was then obtained by correcting for the elastic contribution of the indenter tip using the equation below

$$E_\mathrm{p} = \frac{1-{\nu_\mathrm{p}}^2}{\frac{1}{E_\mathrm{r}} + \frac{1-{\nu_\mathrm{i}}^2}{E_\mathrm{i}}}$$

where E_p is the Young's (elastic) modulus of the polymer; E_r is the reduced modulus; E_i is the elastic modulus (1140 GPa) and ν_i is the Poisson's ratio (0.07) of the diamond Berkovich indenter; and we assume a Poisson's ratio of ν_p =0.35, a typical value for glassy polymers, for the iCVD polymers.[1,3–6]

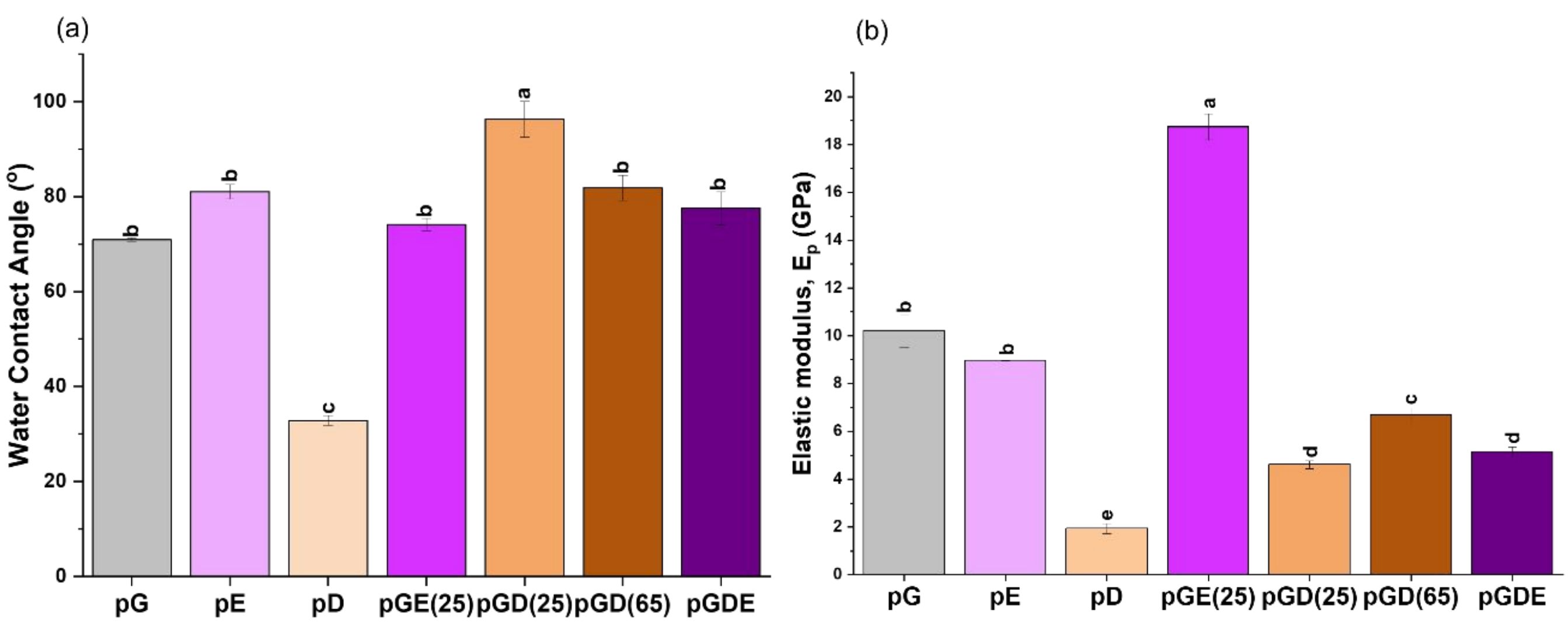

Figure S1. (a) Water contact angle measurements and **(b)** Elastic modulus measured from nanoindentation of different immobilization supports. The means were calculated based on at least three measurements on three separate surface samples. Error bars represent standard error. Non-overlapping letters indicate significant differences across support types (p < 0.05).Click or tap here to enter text.Click or tap here to enter text.

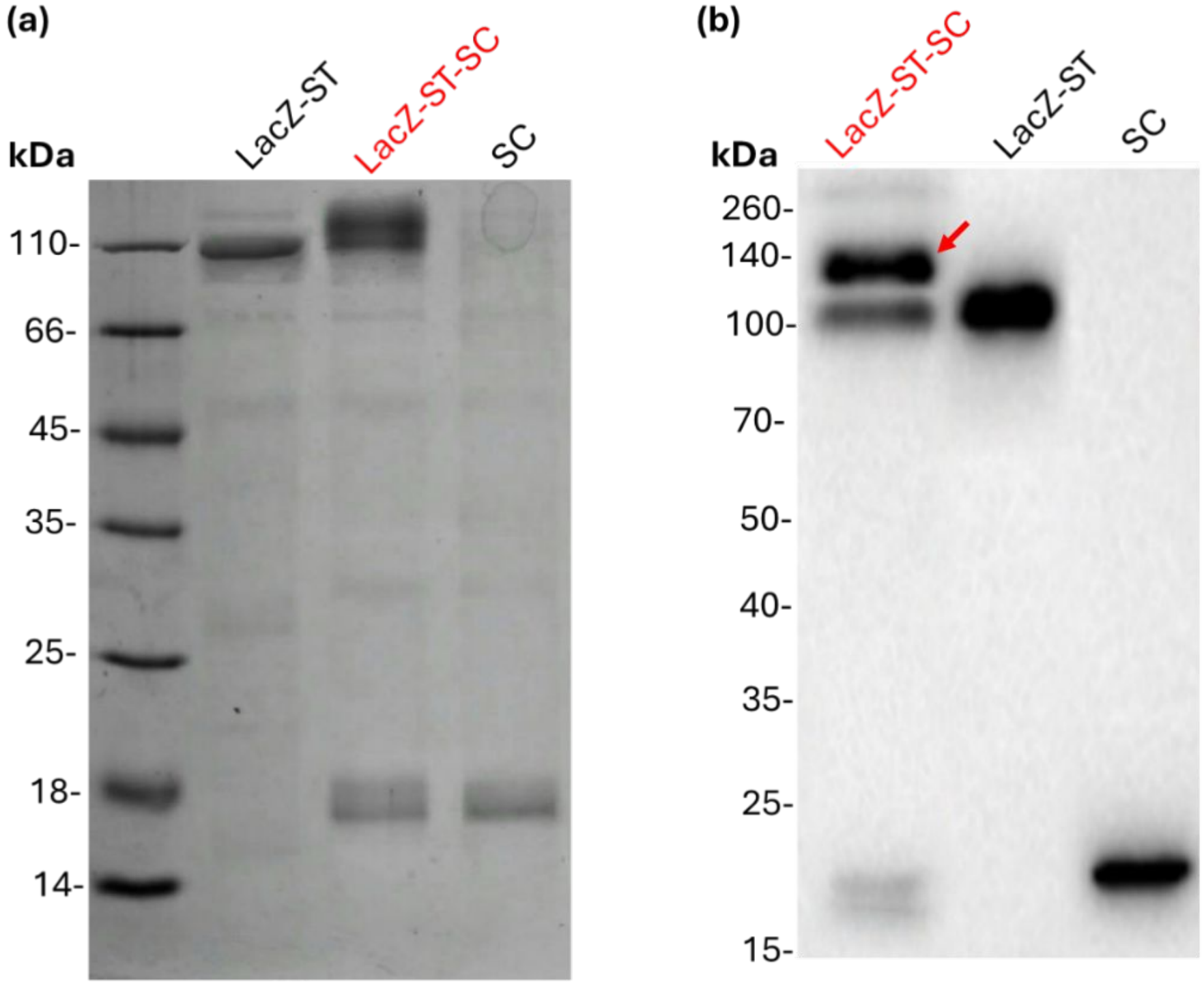

Figure S2. Confirmation of bioconjugation of LacZ-SpyTag (LacZ-ST) to SpyCatcher (SC) in solution. (a) Coomassie blue stain gel of LacZ-ST, LacZ-ST-SC, and SC, purified using the His-Tag on the proteins. (b) Western Blot of the same samples. Blots were probed with HRP-labeled anti-HisTag antibody. The red arrow points to the band corresponding to LacZ-ST-SC. Molecular weights are indicated in kDa.

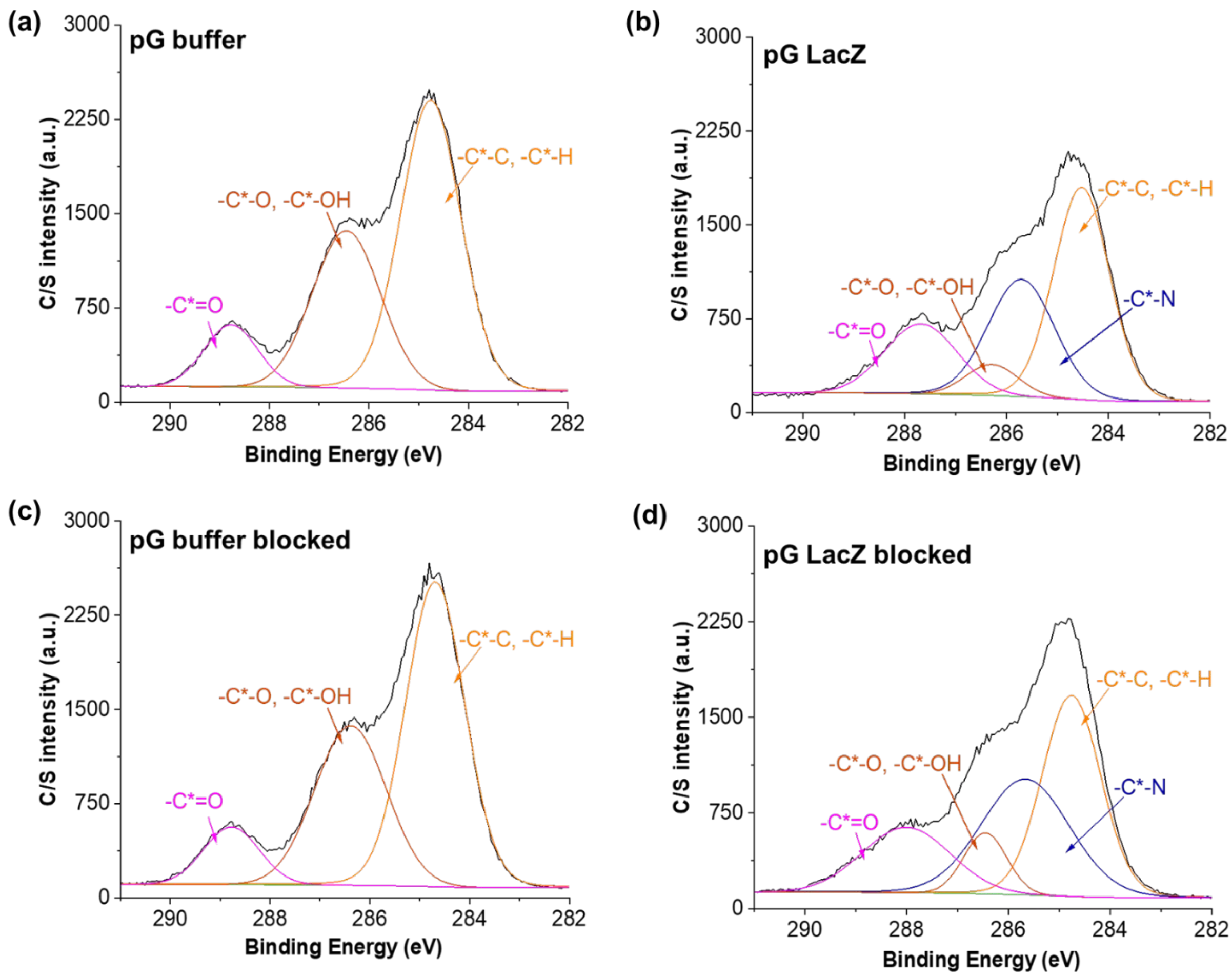

Figure S3. XPS C1s high-resolution spectra of pG samples under various treatment conditions: (a) pG incubated in phosphate buffer (0.1 M, pH 7, 24 h, 37 °C) alone (no LacZ). (b) pG incubated with LacZ following the RI procedure. (c) pG incubated with glycine (1 M) as a blocking agent. (d) pG first incubated with LacZ (as in b), then treated with glycine (1 M).

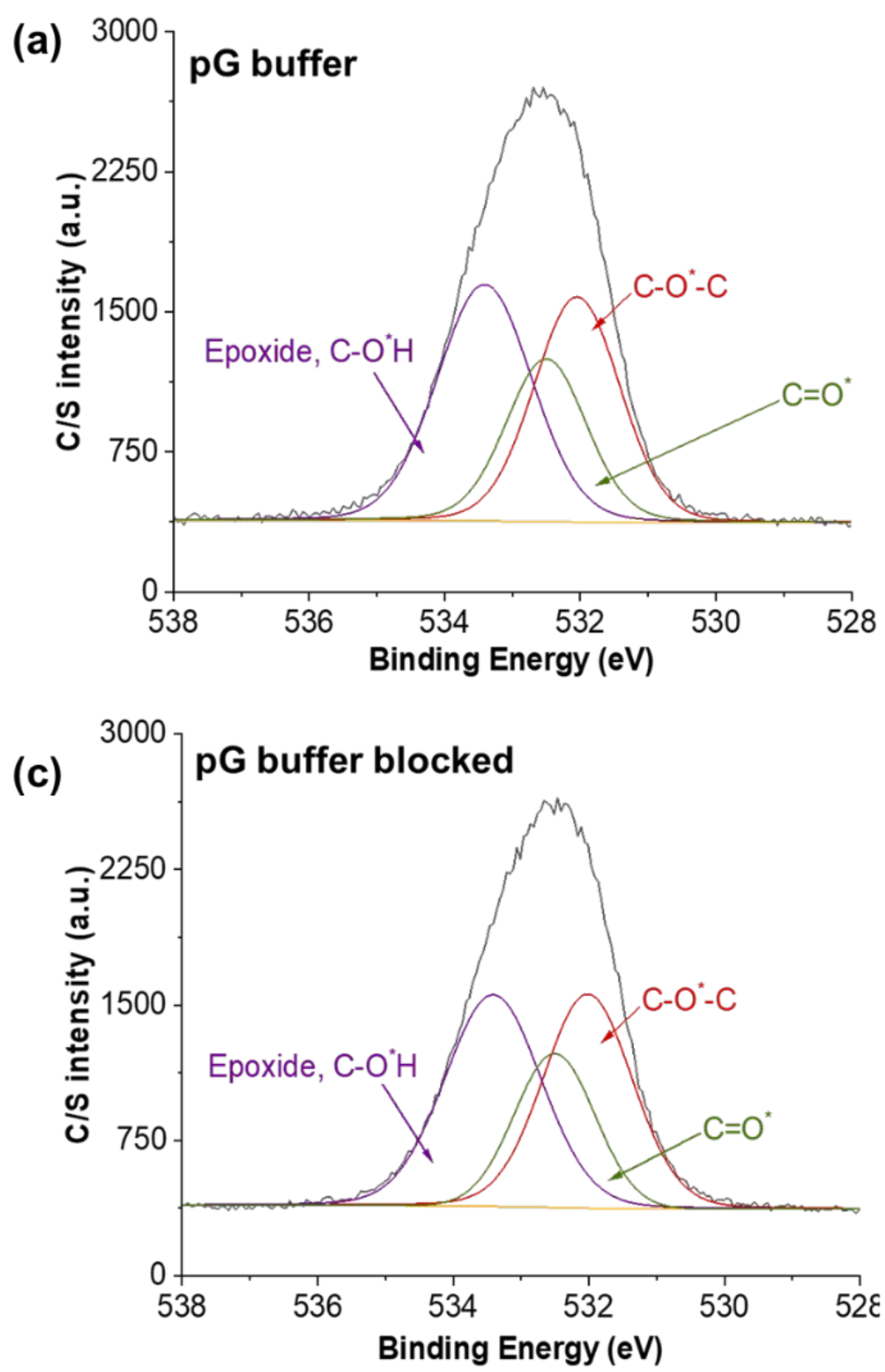

Figure S4. XPS O1s high-resolution spectra of pG samples under various treatment conditions: (a) pG incubated in phosphate buffer (0.1 M, pH 7, 24 h, 37 °C) alone (no LacZ). (b) pG incubated with LacZ following the RI procedure. (c) pG incubated with glycine (1 M) as a blocking agent. (d) pG first incubated with LacZ (as in b), then treated with glycine (1 M).

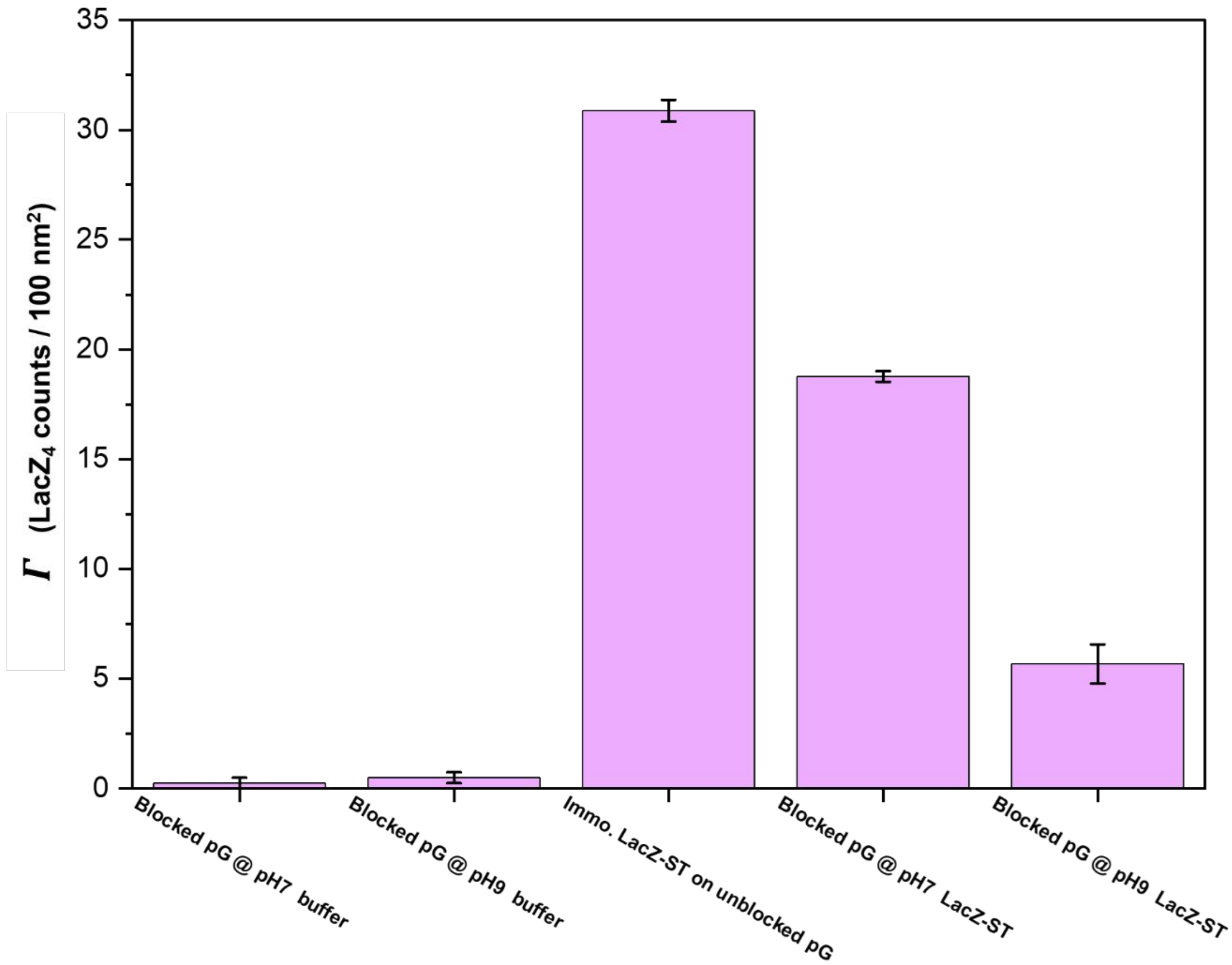

Figure S5. Investigation of the propensity of LacZ-ST to conjugate by way of random immobilization. Blocking of pG supports by glycine (1 M) was carried out at two pHs.

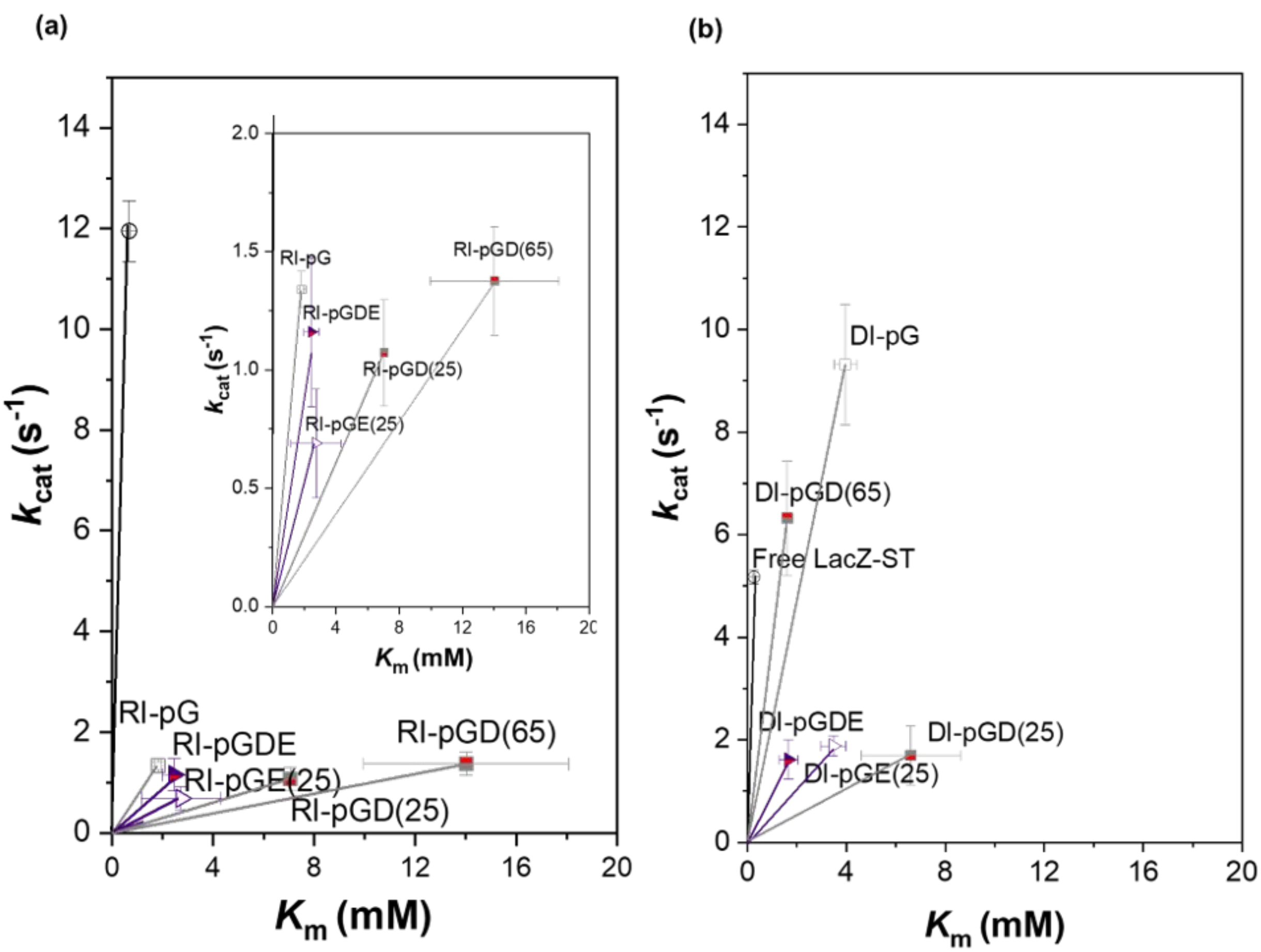

Figure S6. Comparison of Michaelis–Menten kinetic parameters for free and immobilized LacZ at pH 7: (a) LacZ-WT and RI samples (inset shows a magnified view), and (b) LacZ-ST and DI samples. Purple lines indicate supports containing crosslinker (E). Slopes correspond to catalytic efficiency (k_{cat}/K_m).

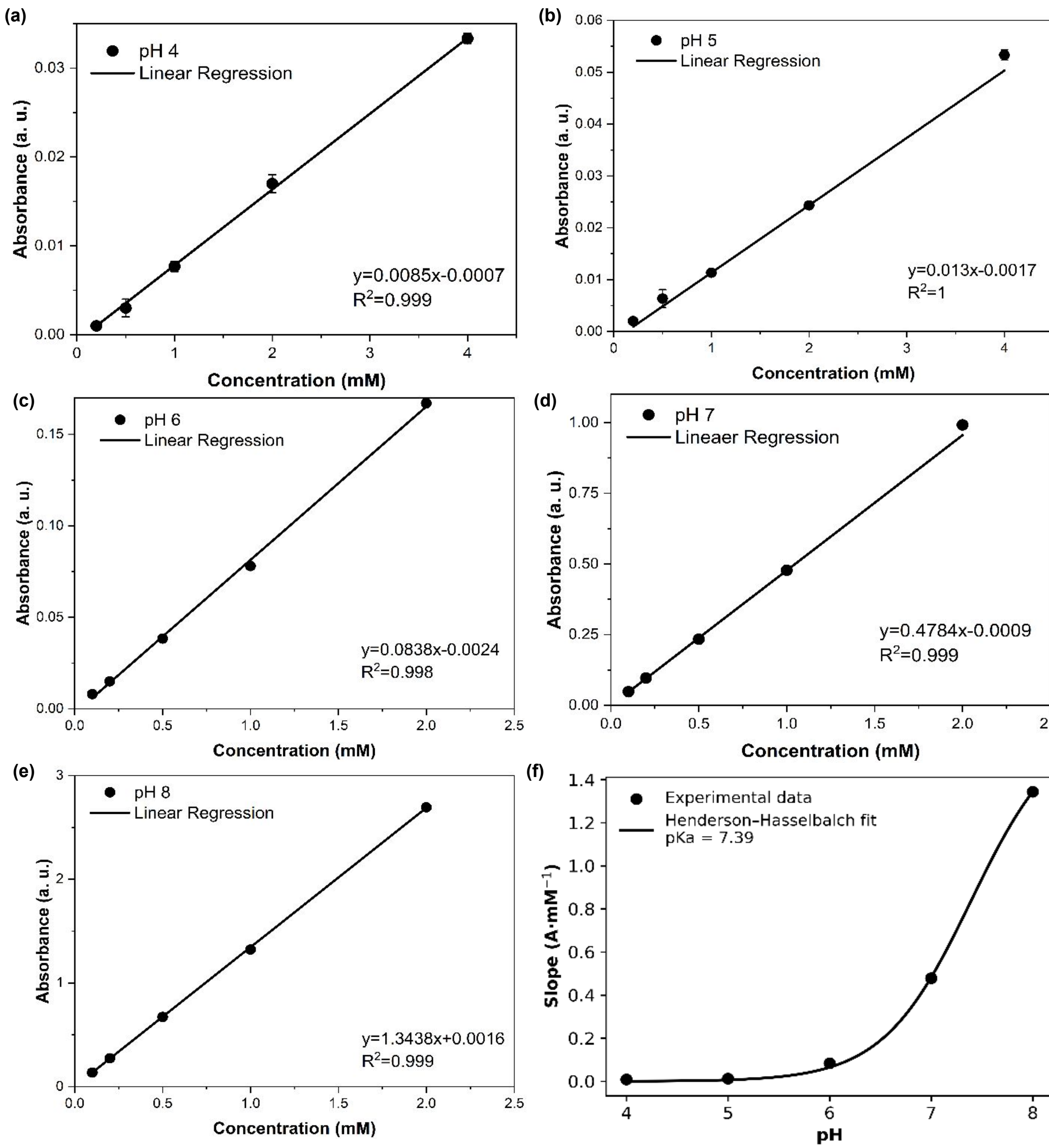

Figure S7. Calibration curves showing ONP absorbance as a function of concentration under buffered conditions at (a) pH 4, (b) pH 5, (c) pH 6, (d) pH 7, and (e) pH 8. Linear regression of each dataset yielded excellent linearity across the tested concentration ranges, with slopes increasing markedly as the solution pH approached and exceeded the ONP ionization region: 0.0085, 0.0130, 0.0838, 0.4784, and 1.3438. (f) The pH-dependent slopes extracted from the calibration curves were fitted using the Henderson–Hasselbalch model, yielding an apparent pK_a of 7.39, which is in good agreement with the reported dissociation constant of ONP in aqueous solution ($pK_a \approx 7.23$ at 25 °C).

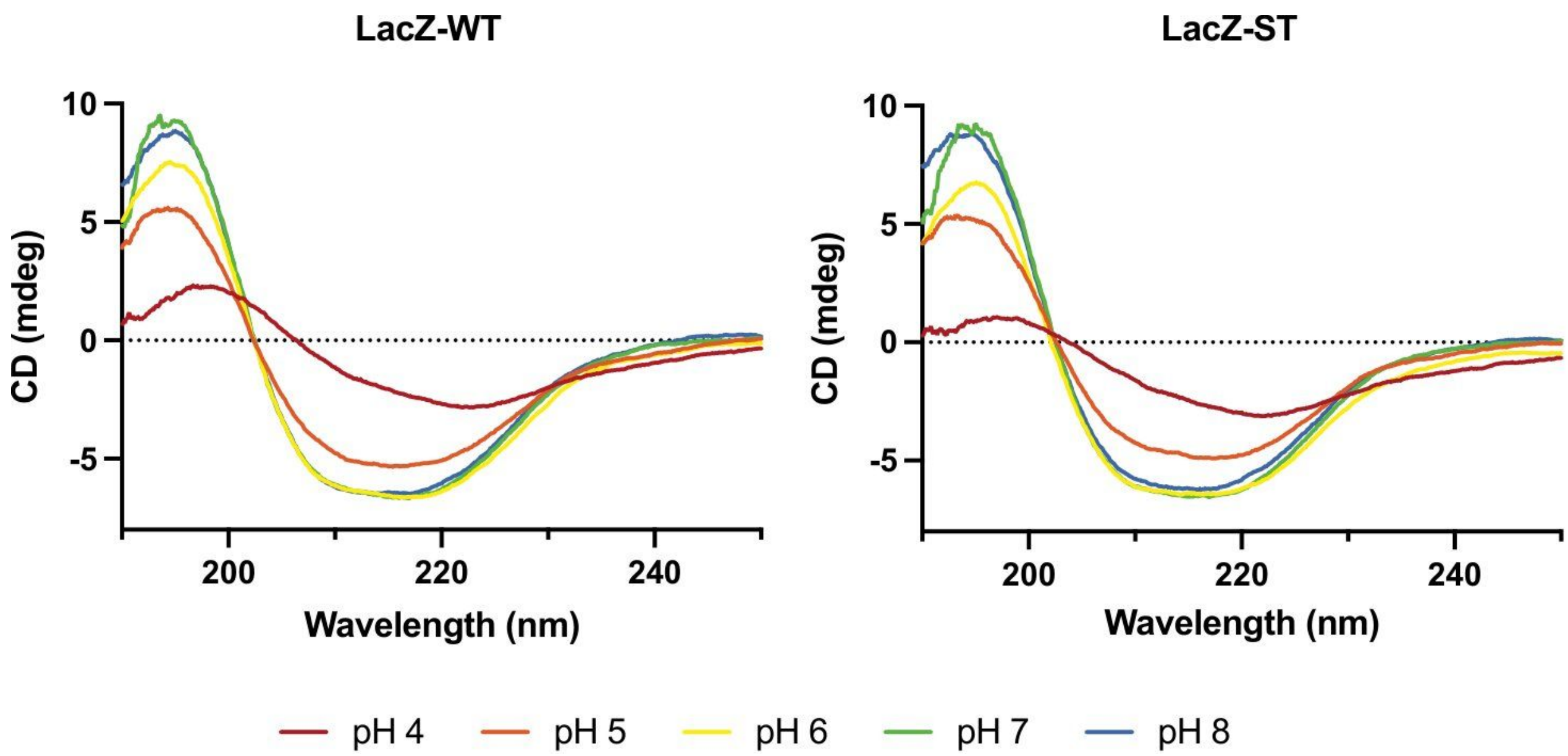

Figure S8. Circular dichroism (CD) spectra of LacZ-WT and LacZ-ST at pH 4 to 8, measured in the wavelength range (190 - 250 nm). Spectral features characteristic of α-helical structures, including a positive band near 195 nm and negative minima at ~208 and ~222 nm, are observed at pH 6 - 8, while reduced ellipticity is evident at pH 4.

Supporting Information

Table S1. Initiated chemical vapor deposition (iCVD) process parameters for synthesizing the enzyme immobilization supports.

**Samples**	**Flow rates (sccm)**					
	**(Co)monomers**			**TBPO**	**Argon**	**Total**
	**G**	**D**	**E**			
**pG**	1.4	NA	NA	0.6	1.0	3.0
**pGE(25)**	0.9	NA	0.1	0.6	1.4	3.0
**pGD(25)**	2.0	0.6	NA	0.6	0.0	3.2
**pGD(65)**	1.0	1.0	NA	0.6	0.4	3.0
**pGDE**	2.0	2.0	0.1	0.6	0.0	4.7

Table S2. Pressure saturation ratios (Pm/Psat) of (co)monomer gases and the resulted deposition rates of polymer thin films

**Sample**	P_m/P_{sat} **(on iCVD stage)**				**Deposition rates (nm min^{-1})***		
	**G**	**D**	**E**	**Total**	DR_{stg}	DR_{plt}	DR_{stg}/DR_{plt}
**pG**	0.16	NA	NA	0.16	19.2	4.0	4.8
**pGE(25)**	0.03	NA	0.1	0.13	2.0	0.4	5.0
**pGD(25)**	0.07	0.07	NA	0.14	96.5	5.7	16.8
**pGD(65)**	0.04	0.13	NA	0.17	46.1	0.5	94.1
**pGDE**	0.05	0.17	0.05	0.27	32.2	7.9	4.1

*DR_{stg} and DR_{plt} denote the deposition rates measured on a Si substrate placed on the iCVD stage and those measured on another Si substrate located atop a 96-well plate, respectively. The 96-well plates were placed on custom-made aluminum plates that fit the shape of the plates well in order to facilitate heat transfer to the iCVD stage.

Table S3 Copolymer molar compositions determined based on transmission FTIR.

	**Copolymer composition (mol%)**		
**Samples**	**G**	**D**	**E**
pGE(25)	74	0	26
pGD(65)	35	65	0
pGD(25)	76	24	0
pGDE	49	37	14

Table S4. Primer information (reproduced from Duan et al 2026 [7])

Primer	Sequences (5' to 3')
LacZ-WT-Nde I-F	aagaaggagatatacatatgaccatgattacggattcactggc
LacZ-LacZ-WT-6xHis-R	caaaacagccaagcttttagtgatggtgatggtgatgtttttgacaccagaccaact
SpyTag-GSlinker-F	tttaagaaggagatatacatatggcgcatatcgtgatggtggatgc
SpyTag-GSlinker-R	ttccggatccacctccggatcctttggtcggtttatacgcatccaccatcacgatatg
pBAD-LacZ-WT-spy-F	gaggtggatccggaagtggtggatccggaagtggatccggaagtatgaccatgattac gg
pBAD-lacz-wt-spy-r	gtatatctccttcttaaagttaaacaaaattatttctagcccaaaaaacgggtatgg
SpyCatcher-Nde I-F	aagaaggagatatacatatggttgataccttatcaggtttatcaagtgagc
SpyCatcher-Hind III-6xHis-R	caaaacagccaagcttttagtgatggtgatggtgatgtaaatatgagcgtcacctttag

Gene Blocks information

SpyCatcher:

5'atggttgataccttatcaggtttatcaagtgagcaaggtcagtccggtgatatgacaattgaagaagatagtgctacccatattaaattc tcaaaacgtgatgaggacggcaaagagttagctggtgcaactatggagttgcgtgattcatctggtaaaactattagtacatggatttca gatggacaagtgaaagatttctacctgtatccaggaaaatatacatttgtcgaaaccgcagcaccagacggttatgaggtagcaactg ctattacctttacagttaatgagcaaggtcaggttactgtaaatggcaaagcaactaaaggtgacgctcatattta3'

Table S5. Random Immobilization (RI) efficiency based on BCA for various immobilization supports used in this study. Values tabulated below are mean ± standard error (N = 3).

Conjugation method—Immobilization Support	No. LacZ-WT /100nm^2 *	IY (%)
**RI-pG**	48.5 ± 4.6	8.6 ± 0.8
**RI-pGE(25)**	43.2 ± 3.0	7.6 ± 0.5
**RI-pGD(25)**	51.0 ± 2.0	9.0 ± 0.4
**RI-pGD(65)**	50.4 ± 6.3	8.9 ± 1.1
**RI-pGDE**	51.0 ± 4.8	9.0 ± 0.9
**RI-SG**	51.3 ± 5.4	9.1 ± 1.0
**RI-SGD(25)**	61.7 ± 5.2	10.9 ± 0.9

* LacZ-WT in tetramer form was used for calculating the population density on surface

Supporting Information

Table S6. Directed Immobilization (DI) efficiency based on BCA for various immobilization supports used in this study. Values tabulated below are mean ± standard error (N = 3).

Conjugation method—Immobilization Support	No. SC /100nm^2	No. LacZ-ST /100nm^2 *	SC occupied (%) **	IY (%)
**DI-pG**	111.3 ± 2.1	9.1 ± 1.4	8.2 ± 1.2	2.5 ± 0.4
**DI-pGE(25)**	128.8 ± 8.2	4.2 ± 1.1	2.9 ± 0.7	1.1 ± 0.3
**DI-pGD(25)**	77.7 ± 5.3	4.2 ± 0.6	5.4 ± 0.8	1.1 ± 0.2
**DI-pGD(65)**	65.8 ± 7.1	4.7 ± 0.5	7.1 ± 0.7	1.3 ± 0.1
**DI-pGDE**	58.8 ± 5.3	9.6 ± 0.4	16.4 ± 0.7	2.6 ± 0.1
**DI-SG**	126.0 ± 9.5	5.4 ± 0.7	4.2 ± 0.6	1.4 ± 0.2
**DI-SGD(25)**	126.7 ± 12.4	9.4 ± 0.9	7.4 ± 0.7	2.5 ± 0.2

* LacZ-ST in tetramer form was used for calculating the population density on surface

** “SC occupied (%)” is the percentage of SpyCatcher occupied by LacZ-ST tetramers, computed using the following formular: $SC\ occupied\ (\%) = \frac{No.\ LacZ{-}ST(teramers)}{No.\ SC} \times \mathbf{100}$

Table S7. Kinetic Parameters of Randomly Immobilized LacZ-WT at pH 7 represented as mean ± standard error. Non-overlapping letters represent significant differences within the same column ($p < 0.05$; N = 3).

Sample	V_{max} (mM s^{-1})*	K_m (mM)	k_{cat} (s^{-1})**	k_{cat}/K_m (mM^{-1} s^{-1})
**Surface Free**	4.48E-03 ± 0.12E-03[a]	0.67 ± 0.03[e]	11.95 ± 0.34[a]	17.96 ± 1.04[a]
**RI-pG**	3.85E-05 ± 0.25E-05[b]	1.82 ± 0.13[d]	1.34 ± 0.09[b]	0.74 ± 0.18[c]
**RI-pGE(25)**	1.75E-05 ± 0.61E-05[b]	2.73 ± 1.57[cd]	0.69 ± 0.24[c]	0.25 ± 0.19[de]
**RI-pGD(25)**	3.23E-05 ± 0.68E-05[b]	7.05 ± 0.20[b]	1.07 ± 0.23[bc]	0.15 ± 0.03[e]
**RI-pGD(65)**	4.09E-05 ± 0.68E-05[b]	14.02 ± 4.06[a]	1.38 ± 0.22[b]	0.10 ± 0.03[e]
**RI-pGDE**	3.50E-05 ± 0.95E-05[b]	2.45 ± 0.48[cd]	1.16 ± 0.31[bc]	0.47 ± 0.09[cd]
**RI-SG**	3.16E-05 ± 1.40E-05[b]	0.81 ± 0.35[e]	1.79 ± 0.43[b]	2.21 ± 0.33[b]
**RI-SGD(25)**	4.68E-05 ± 0.42E-05[b]	1.46 ± 0.41[de]	1.29 ± 0.11[b]	0.88 ± 0.32[c]

*V_{max} was calculated based on o-nitrophenol produced by LacZ catalysis.

**k_{cat} was calculated by normalizing V_{max} with [$LacZ_{monomer}$].

Table S8. Kinetic Parameters of Randomly Immobilized LacZ-WT at pH 4 represented as mean ± standard error. Non-overlapping letters represent significant differences within the same column ($p < 0.05$; N = 3).

Sample	V_{max} (mM s^{-1})*	K_m (mM)	k_{cat} (s^{-1})**	k_{cat}/K_m (mM^{-1} s^{-1})
**LacZ-WT**	ND	ND	ND	ND
**RI-pG**	ND	ND	ND	ND
**RI-pGE(25)**	ND	ND	ND	ND
**RI-pGD(25)**	1.51E-05 ± 0.63E-05[a]	19.33 ± 4.24[a]	0.58 ± 0.12[a]	0.03 ± 0.00[c]
**RI-pGD(65)**	9.37E-06 ± 0.56E-06[b]	6.94 ± 1.75[b]	0.32 ± 0.02[b]	0.05 ± 0.01[b]
**RI-pGDE**	7.36E-06 ± 1.82E-06[bc]	1.30 ± 0.24[c]	0.24 ± 0.06[bc]	0.18 ± 0.01[a]
**RI-SG**	ND	ND	ND	ND
**RI-SGD(25)**	4.70E-06 ± 1.28E-06[c]	3.16 ± 0.79[bc]	0.13 ± 0.04[c]	0.04 ± 0.00[bc]

*V_{max} was calculated based on o-nitrophenol produced by LacZ catalysis.

**k_{cat} was calculated by normalizing V_{max} with [$LacZ_{monomer}$]

Supporting Information

Table S9. Kinetic Parameters of Directedly Immobilized LacZ-ST at pH 7 represented as mean $\pm$ standard error. Non-overlapping letters represent significant differences within the same column ($p < 0.05$; N = 3).

Sample	V_{max} (mM s^{-1})	K_m (mM)	k_{cat} (s^{-1})	k_{cat}/K_m (mM^{-1} s^{-1})
**Surface Free**	1.94E-03 $\pm$ 0.05E-03[a]	0.27 $\pm$ 0.02[d]	5.18 $\pm$ 0.14[b]	18.99 $\pm$ 1.28[a]
**DI-pG**	1.79E-05 $\pm$ 0.42E-05[b]	3.96 $\pm$ 0.46[bc]	9.32 $\pm$ 1.18[a]	2.36 $\pm$ 0.08[b]
**DI-pGE(25)**	5.60E-06 $\pm$ 0.57E-06 [b]	3.47 $\pm$ 0.52[c]	1.88 $\pm$ 0.19[d]	0.54 $\pm$ 0.03[de]
**DI-pGD(25)**	1.74E-05 $\pm$ 0.58E-05 [b]	6.61 $\pm$ 2.01[a]	1.70 $\pm$ 0.58[d]	0.26 $\pm$ 0.09[e]
**DI-pGD(65)**	2.23E-05 $\pm$ 0.39E-05 [b]	1.61 $\pm$ 0.23[d]	6.33 $\pm$ 1.12[ab]	3.93 $\pm$ 1.52[b]
**DI-pGDE**	1.65E-05 $\pm$ 0.40E-05 [b]	1.66 $\pm$ 0.38[d]	1.61 $\pm$ 0.38[d]	0.97 $\pm$ 0.15[c]
**DI-SG**	1.30E-05 $\pm$ 1.01E-08 [b]	5.28 $\pm$ 0.55[ab]	1.70 $\pm$ 0.00[d]	0.32 $\pm$ 0.03[e]
**DI-SGD(25)**	2.34E-05 $\pm$ 0.72E-05 [b]	4.33 $\pm$ 2.33[abc]	3.31 $\pm$ 1.01[c]	0.77 $\pm$ 0.18[cd]

*V_{max} was calculated based on o-nitrophenol produced by LacZ catalysis.

**k_{cat} was calculated by normalizing V_{max} with [$LacZ_{monomer}$]

Supporting Information

Table S10. Kinetic Parameters of Directedly Immobilized LacZ-ST at pH 4 represented as mean ± standard error. Non-overlapping letters represent significant differences within the same column ($p < 0.05$; N = 3).

**Sample**	V_{max} **(mM s^{-1})***	K_m **(mM)**	k_{cat} **(s^{-1})****	k_{cat}/K_m **(mM^{-1} s^{-1})**
**LacZ-ST**	ND	ND	ND	ND
**DI-pG**	ND	ND	ND	ND
**DI-pGE(25)**	ND	ND	ND	ND
**DI-pGD(25)**	3.31E-06 ± 0.22E-06 [b]	12.40 ± 1.03[ab]	0.32 ± 0.02[c]	0.03 ± 0.00[c]
**DI-pGD(65)**	3.25E-06 ± 0.05E-06 [b]	13.85 ± 0.72[ab]	0.92 ± 0.01[b]	0.07 ± 0.00[b]
**DI-pGDE**	1.03E-05 ± 0.11E-05 [a]	11.53 ± 4.01[b]	0.99 ± 0.11[b]	0.09 ± 0.02[ab]
**DI-SG**	ND	ND	ND	ND
**DI-SGD(25)**	1.12E-05 ± 0.11E-05 [a]	16.44 ± 0.07[a]	1.59 ± 0.15[a]	0.10 ± 0.01[a]

*V_{max} was calculated based on o-nitrophenol produced by LacZ catalysis.

**k_{cat} was calculated by normalizing V_{max} with [$LacZ_{monomer}$]

Table S11. Thickness and penetration depth of tip used in nanoindentation represented as mean ± standard deviation

Polymer	Film Thickness (nm)	Penetration Depth of Tip (nm)	Depth / Thickness (%)
**pG**	241.24 ± 0.38	135.44 ± 8.00	56.14
**pE**	366.25 ± 8.22	95.43 ± 0.70	26.06
**pD**	1904.70 ± 70.22	238.29 ± 25.78	12.51
**pGE(25)**	270.16 ± 9.17	103.40 ± 2.08	38.27
**pGD(25)**	907.20 ± 12.91	148.55 ± 5.42	16.37
**pGD(65)**	1059.55 ± 17.17	113.44 ± 7.35	10.71
**pGDE**	289.46 ± 13.19	261.32 ± 13.68	90.28

References

(1) Oliver, W. C.; Pharr, G. M. An Improved Technique for Determining Hardness and Elastic Modulus Using Load and Displacement Sensing Indentation Experiments. *J. Mater. Res.* **1992**, *7* (6), 1564–1583. https://doi.org/10.1557/jmr.1992.1564.
(2) Oliver, W. C.; Pharr, G. M. Measurement of Hardness and Elastic Modulus by Instrumented Indentation: Advances in Understanding and Refinements to Methodology. *J. Mater. Res.* **2004**.
(3) Winer, W. O.; Bergles, A. E.; Klutke, G. A.; Wang, K. K.; Finnie, I.; Welty, J. R.; Bryant, M. D.; Yang, H. T.; Mow, V. C.; Leckie, F. A.; Gross, D. *Mechanical Engineering Series Series Editors*, Third Edition.; Ling, F. F., Ed.; Springer Science+Business Media, 2011.
(4) Arora, G.; Pathak, H. Nanoindentation Characterization of Polymer Nanocomposites for Elastic and Viscoelastic Properties: Experimental and Mathematical Approach. *Composites Part C: Open Access* **2021**, *4*. https://doi.org/10.1016/j.jcomc.2020.100103.
(5) Charitidis, C. Nanomechanical Properties and Nanoscale Deformation of Engineering Metals and Alloys. *Materials and Surface Engineering: Research and Development* **2012**, 23–59. https://doi.org/10.1533/9780857096036.23.
(6) Zhang, Q. Multi-Scale Instrumented Indentation of Hydrogels. *The Mechanics of Hydrogels: Mechanical Properties, Testing, and Applications* **2022**, 91–118. https://doi.org/10.1016/B978-0-08-102862-9.00001-4.
(7) Duan, H.; Chen, J.; Fianu, F.; Sun, W.; Cheng, Y. Nanoscale Protonation Limits and Charge Density in Polymer Films Govern the Activity of Immobilized LacZ under Acid Stress. *Nanoscale* **2026**. https://doi.org/10.1039/D5NR05409E.
